# Supplementary material for: The Potential Public Health Impact of the mRNA-Based Respiratory Syncytial Virus Vaccine, mRNA-1345, Under Extended Vaccination Campaigns Among Older Adults in the United Kingdom: A Modelling Study
Source: Vaccines (Basel). 2025 Oct 18;13(10):1065. doi: 10.3390/vaccines13101065 (PMC12567615; doi:10.3390/vaccines13101065)
Supplement: Supplementary file 1 [file vaccines-13-01065-s001.zip › vaccines-3873899-supplementary.pdf]

Supplementary materials

Manuscript: The Potential Public Health Impact of the mRNA-Based Respiratory Syncytial Virus Vaccine, mRNA-1345, Under Extended Vaccination Campaigns Among Older Adults in the United Kingdom: A Modelling Study

S1 Supplement 1: Detailed methodology and inputs

S1.1 Modelling methodology

S1.1.1 Model compartments

While the full model includes compartments for both vaccinated and unvaccinated individuals to capture differential infection risks and disease progression, an additional version excluding vaccination-related compartments was used during model calibration and simulations for strategy without vaccination. This approach minimized computational burden while preserving core transmission dynamics. **Table S1** provides a brief description of the model compartments.

Table S1. Description of the epidemiological compartments of the model.

Compartment	Descriptions
	Main compartments
M	Individuals completely protected from RSV infection due to maternal immunity
S_i for $i \epsilon (1,2,3,4)$	Individuals susceptible to RSV infection, before i-th infection
E_i for $i \epsilon (1,2,3,4)$	Individuals infected by RSV, but not yet infectious (i.e., exposed), who have experienced i infections (including a current one)
A_i for $i \epsilon (1,2,3,4)$	Individuals infected by RSV, infectious, and asymptomatic, who have experienced i infections (including a current one)
I_i for $i \epsilon (1,2,3,4)$	Individuals infected by RSV, infectious, and symptomatic (with ARD), who have experienced i infections (including a current one)
R_i for $i \epsilon (1,2,3,4)$	Individuals recovered from RSV infection, completely protected from a new infection due to natural immunity, who have experienced i previous infections
V_4^j for j $\epsilon (1, \ldots ,157)$	Vaccinated individuals, who have experienced 3+ previous infections, j-th week since vaccination
E_{V4}	Vaccinated individuals, infected by RSV, but not yet infectious (i.e., exposed), who have experienced 4+ infections (including a current one)
A_{V4}	Vaccinated individuals, infected by RSV, infectious, and asymptomatic, who have experienced 4+ infections (including a current one)
I_{V4}	Vaccinated individuals, infected by RSV, infectious, and symptomatic (with ARD), who have experienced 4+ infections (including a current one)
	Observational compartments
INF	Total number of RSV infections (symptomatic and asymptomatic)
ARD	Number of ARD cases
L	Number of LRTD cases
H	Number of hospitalized cases due to LRTD
D	Number of fatal cases due to hospitalized LRTD

Each of the model compartments was stratified into 5-year age groups before 85 years, and a group ≥85 years.

ARD, acute respiratory disease; LRTD, lower respiratory tract disease; RSV, respiratory syncytial virus.

S1.1.2 Model parameters

Ordinary differential equations (ODE) and additional calculations were parameterized to provide a mathematical notation. A list of parameters used in the model calculations is presented in **Table S2**.

Table S2. Parameters used in the differential equations.

Parameter	Type	Description
		Meta-parameters
q	Variable	Indicates whether transmission type depends on population density or not (density-dependent transmission or frequency-dependent transmission, 0 or 1 respectively):
a, k	Variable	Age group, according to age stratification in the model
t	Variable	Time point, in weeks
		Demographic parameters
$N(t)$	Calculated within ODE	Total population size at time t
$N(a,t)$	Calculated within ODE	Population size in age group a, at time t
$\xi(a)$	Calculated within ODE	Ageing rate from age group a to age group $a+1$
c_{ak}	Input	Number of contacts between a person from age group a and a person from age group k
$\mu_{pop}(a)$	Input	Rate of change for the population size, including general mortality and migration in population, for age group a
$birth$	Input	The rate at which individuals enter the model
		Epidemiological parameters required to estimate the force of infection (FOI)
$\lambda(a,t)$	Calculated within ODE	FOI in age group a, at time t
$\beta_{a,k}$	Calculated within ODE	Transmission rate from age group k to age group a
b	Estimated in calibration	Transmission probability (transmission probability from infectious to susceptible individual)
s_{amp}	Estimated in calibration	Seasonality parameter, amplitude of sinusoidal function
s_{shift}	Estimated in calibration	Seasonality parameter, horizontal shift of sinusoidal function
ψ_i for $i \in (1,2,3,4)$	Input	Relative infectiousness of i-th infection, equal to 1 for i=1
		Other epidemiological parameters
ε	Input	Rate of becoming infectious
γ_i for $i \in (1,2,3,4)$	Input	Recovery rate from i-th infection
σ_i for $i \in (1,2,3)$	Input	Relative risk of re-infection, after i-th infection
$p(a)$	Input	Proportion of infections that are asymptomatic, for age group a
$\kappa_i(a)$ for $i \in (1,2,3,4)$	Input	Proportion of ARD infections that are LRTDs, for age group a, for i-th infection
$\delta_i(a)$ for $i \in (1,2,3,4)$	Input	Proportion of hospitalisations due to LRTDs, for age group a, for i-th infection
$\mu(a)$	Input	Proportion of deaths due to hospitalized RSV, for age group a
η_m	Input	Waning rate of maternal immunity
η_i for $i \in (1,2,3,4)$	Input	Waning rate of post-infection immunity, for i-th infection
		Vaccine related parameters
$\chi_{j,t_{RV}}$	Variable	Parameter which indicates whether the individual in V_4^j compartment is revaccinated (1) or not (0); equal 1 when j > t_{RV}, 0 when j ≤ t_{RV}, in j-th week since last vaccination
t_{RV}	Input	Number of weeks after which individual with previous vaccination can be revaccinated
$\theta(a,t)$	Input	Vaccination coverage for age group a at time t
σ_4^j	Input	Relative risk of RSV infection in vaccinated versus unvaccinated individuals, in j-th week since vaccination
σ_5^j	Input	Relative risk of LRTD given ARD in vaccinated versus unvaccinated individuals, in j-th week since vaccination
σ_6^j	Input	Relative risk of hospitalisation given LRTD in vaccinated versus unvaccinated individuals, in j-th week since vaccination
γ_{V4}	Input	Recovery rate from 4+ infection for a vaccinated individual

ARD, acute respiratory disease; FOI, force of infection; LRTD, lower respiratory tract disease; ODE, ordinary differential equations; RSV, respiratory syncytial virus.

S1.1.3 Differential equations

The ODEs used in the model are provided below, for age group a are at time t:

Compartments for individuals with no previous respiratory syncytial virus (RSV) infection

$$\frac{dM(a,t)}{dt} = birth \cdot N(t) - M(a,t) \cdot (\eta_m + \mu_{pop}(a)) - M(a,t) \cdot \xi(a) + M(a-1,t) \cdot \xi(a-1)$$

$$\frac{dS_1(a,t)}{dt} = M(a,t) \cdot \eta_m - S_1(a,t) \cdot (\lambda(a,t) + \mu_{pop}(a)) - S_1(a,t) \cdot \xi(a) + S_1(a-1,t) \cdot \xi(a-1)$$

$$\frac{dE_1(a,t)}{dt} = S_1(a,t) \cdot \lambda(a,t) - E_1(a,t) \cdot (\varepsilon + \mu_{pop}(a)) - E_1(a,t) \cdot \xi(a) + E_1(a-1,t) \cdot \xi(a-1)$$

$$\frac{dA_1(a,t)}{dt} = E_1(a,t) \cdot \varepsilon \cdot p(a) - A_1(a,t) \cdot (\gamma_1 + \mu_{pop}(a)) - A_1(a,t) \cdot \xi(a) + A_1(a-1,t) \cdot \xi(a-1)$$

$$\frac{dI_1(a,t)}{dt} = E_1(a,t) \cdot \varepsilon \cdot (1 - p(a)) - I_1(a,t) \cdot \left(\gamma_1 + \mu_{pop}(a)\right) - I_1(a,t) \cdot \xi(a) + I_1(a-1,t) \cdot \xi(a-1)$$

$$\frac{dR_1(a,t)}{dt} = (A_1(a,t) + I_1(a,t)) \cdot \gamma_1 - R_1(a,t) \cdot \left(\eta_1 + \mu_{pop}(a)\right) - R_1(a,t) \cdot \xi(a) + R_1(a-1,t) \cdot \xi(a-1)$$

Compartments for individuals with one previous RSV infection

$$\frac{dS_2(a,t)}{dt} = R_1(a,t) \cdot \eta_1 - S_2(a,t) \cdot (\lambda(a,t) \cdot \sigma_1 + \mu_{pop}(a)) - S_2(a,t) \cdot \xi(a) + S_2(a-1,t) \cdot \xi(a-1)$$

$$\frac{dE_2(a,t)}{dt} = S_2(a,t) \cdot \lambda(a,t) \cdot \sigma_1 - E_2(a,t) \cdot (\varepsilon + \mu_{pop}(a)) - E_2(a,t) \cdot \xi(a) + E_2(a-1,t) \cdot \xi(a-1)$$

$$\frac{dA_2(a,t)}{dt} = E_2(a,t) \cdot \varepsilon \cdot p(a) - A_2(a,t) \cdot (\gamma_2 + \mu_{pop}(a)) - A_2(a,t) \cdot \xi(a) + A_2(a-1,t) \cdot \xi(a-1)$$

$$\frac{dI_2(a,t)}{dt} = E_2(a,t) \cdot \varepsilon \cdot (1 - p(a)) - I_2(a,t) \cdot \left(\gamma_2 + \mu_{pop}(a)\right) - I_2(a,t) \cdot \xi(a) + I_2(a-1,t) \cdot \xi(a-1)$$

$$\frac{dR_2(a,t)}{dt} = (A_2(a,t) + I_2(a,t)) \cdot \gamma_2 - R_2(a,t) \cdot \left(\eta_2 + \mu_{pop}(a)\right) - R_2(a,t) \cdot \xi(a) + R_2(a-1,t) \cdot \xi(a-1)$$

Compartments for individuals with two previous RSV infections

$$\frac{dS_3(a,t)}{dt} = R_2(a,t) \cdot \eta_2 - S_3(a,t) \cdot (\lambda(a,t) \cdot \sigma_2 + \mu_{pop}(a)) - S_3(a,t) \cdot \xi(a) + S_3(a-1,t) \cdot \xi(a-1)$$

$$\frac{dE_3(a,t)}{dt} = S_3(a,t) \cdot \lambda(a,t) \cdot \sigma_2 - E_3(a,t) \cdot (\varepsilon + \mu_{pop}(a)) - E_3(a,t) \cdot \xi(a) + E_3(a-1,t) \cdot \xi(a-1)$$

$$\frac{dA_3(a,t)}{dt} = E_3(a,t) \cdot \varepsilon \cdot p(a) - A_3(a,t) \cdot (\gamma_3 + \mu_{pop}(a)) - A_3(a,t) \cdot \xi(a) + A_3(a-1,t) \cdot \xi(a-1)$$

$$\frac{dI_3(a,t)}{dt} = E_3(a,t) \cdot \varepsilon \cdot (1 - p(a)) - I_3(a,t) \cdot \left(\gamma_3 + \mu_{pop}(a)\right) - I_3(a,t) \cdot \xi(a) + I_3(a-1,t) \cdot \xi(a-1)$$

$$\frac{dR_3(a,t)}{dt} = (A_3(a,t) + I_3(a,t)) \cdot \gamma_3 - R_3(a,t) \cdot \left(\eta_3 + \mu_{pop}(a)\right) - R_3(a,t) \cdot \xi(a) + R_3(a-1,t) \cdot \xi(a-1)$$

Compartments for individuals with three or more previous RSV infections

$$\frac{dS_4(a,t)}{dt} = R_3(a,t) \cdot \eta_3 + R_4(a,t) \cdot \eta_4 - S_4(a,t) \cdot \left(\lambda(a,t) \cdot \sigma_3 - \theta(a,t) + \mu_{pop}(a)\right) - S_4(a,t) \cdot \xi(a) + S_4(a-1,t) \cdot \xi(a-1)$$

$$\frac{dV_4^1(a,t)}{dt} = S_4(a,t) \cdot \theta(a,t) + \sum_{j=t_{RV}+1}^{157} V_4^j(a,t) \cdot \theta(a,t) - V_4^1(a,t)$$

$$\frac{dV_4^j(a,t)}{dt} = V_4^{j-1}(a,t) \cdot \left(1 - \lambda(a,t) \cdot \sigma_3 \cdot \sigma_4^{j-1} - \chi_{j-1,t_{RV}} \cdot \theta(a,t) - \mu_{pop}(a)\right) - V_4^{j-1}(a,t) \cdot \left(1 - \lambda(a,t) \cdot \sigma_3 \cdot \sigma_4^{j-1} - \chi_{j-1,t_{RV}} \cdot \theta(a,t) - \mu_{pop}(a)\right) \cdot \xi(a) + V_4^{j-1}(a-1,t) \cdot \left(1 - \lambda(a-1,t) \cdot \sigma_3 \cdot \sigma_4^{j-1} - \chi_{j-1,t_{RV}} \cdot \theta(a-1,t) - \mu_{pop}(a-1)\right) \cdot \xi(a-1) - V_4^j(a,t), 1 < j < 157$$

$$\frac{dV_4^{157}(a,t)}{dt} = V_4^{156}(a,t) \cdot \left(1 - \lambda(a,t) \cdot \sigma_3 \cdot \sigma_4^{156} - \chi_{156,t_{RV}} \cdot \theta(a,t) - \mu_{pop}(a)\right) - V_4^{156}(a,t) \cdot \left(1 - \lambda(a,t) \cdot \sigma_3 \cdot \sigma_4^{156} - \chi_{156,t_{RV}} \cdot \theta(a,t) - \mu_{pop}(a)\right) \cdot \xi(a) + V_4^{156}(a-1,t) \cdot \left(1 - \lambda(a-1,t) \cdot \sigma_3 \cdot \sigma_4^{156} - \chi_{156,t_{RV}} \cdot \theta(a-1,t) - \mu_{pop}(a-1)\right) \cdot \xi(a-1) - V_4^{157}(a,t) \cdot \left(\lambda(a,t) \cdot \sigma_3 \cdot \sigma_4^{157} + \chi_{157,t_{RV}} \cdot \theta(a,t) + \mu_{pop}(a)\right) - V_4^{157}(a,t) \cdot \xi(a) + V_4^{157}(a-1,t) \cdot \xi(a-1)$$

$$\frac{dE_4(a,t)}{dt} = S_4(a,t) \cdot \lambda(a,t) \cdot \sigma_3 - E_4(a,t) \cdot \left(\varepsilon + \mu_{pop}(a)\right) - E_4(a,t) \cdot \xi(a) + E_4(a-1,t) \cdot \xi(a-1)$$

$$\frac{dE_{V4}(a,t)}{dt} = \sum_{j=1}^{157} V_4^j(a,t) \cdot \lambda(a,t) \cdot \sigma_3 \cdot \sigma_4^j - E_{V4}(a,t) \cdot \left(\varepsilon + \mu_{pop}(a)\right) - E_{V4}(a,t) \cdot \xi(a) + E_{V4}(a-1,t) \cdot \xi(a-1)$$

$$\frac{dA_4(a,t)}{dt} = E_4(a,t) \cdot \varepsilon \cdot p(a) - A_4(a,t) \cdot \left(\gamma_4 + \mu_{pop}(a)\right) - A_4(a,t) \cdot \xi(a) + A_4(a-1,t) \cdot \xi(a-1)$$

$$\frac{dA_{V4}(a,t)}{dt} = E_{V4}(a,t) \cdot \varepsilon \cdot p(a) - A_{V4}(a,t) \cdot \left(\gamma_{V4} + \mu_{pop}(a)\right) - A_{V4}(a,t) \cdot \xi(a) + A_{V4}(a-1,t) \cdot \xi(a-1)$$

$$\frac{dI_4(a,t)}{dt} = E_4(a,t) \cdot \varepsilon \cdot \left(1 - p(a)\right) - I_4(a,t) \cdot \left(\gamma_4 + \mu_{pop}(a)\right) - I_4(a,t) \cdot \xi(a) + I_4(a-1,t) \cdot \xi(a-1)$$

$$\frac{dI_{V4}(a,t)}{dt} = E_{V4}(a,t) \cdot \varepsilon \cdot \left(1 - p(a)\right) - I_{V4}(a,t) \cdot \left(\gamma_{V4} + \mu_{pop}(a)\right) - I_{V4}(a,t) \cdot \xi(a) + I_{V4}(a-1,t) \cdot \xi(a-1)$$

$$\frac{dR_4(a,t)}{dt} = \left(A_4(a,t) + I_4(a,t)\right) \cdot \gamma_4 + \left(A_{V4}(a,t) + I_{V4}(a,t)\right) \cdot \gamma_{V4} - R_4(a,t) \cdot \left(\eta_4 + \mu_{pop}(a)\right) - R_4(a,t) \cdot \xi(a) + R_4(a-1,t) \cdot \xi(a-1)$$

S1.1.4 Force of infection

The force of infection defines the risk of acquiring RSV for a susceptible individual, i.e., a transition from compartment S (susceptible) to compartment E (exposed).

It was assumed that the probability of transmission is seasonal, and it was modelled using a cosine function.

The chance of being infected for susceptible individuals in the age group a depends on their contacts with infectious individuals in population. The transmission rate from age group k to age group a, denoted $\beta_{a,k}$, is estimated using the age-specific number of contacts between age group a and k per time step ($c_{a,k}$) and the probability of transmission given contact between an infectious and a susceptible individual (b). Parameter q indicates whether transmission depends or not on the population size (q equal to 0 or 1, respectively), with q assumed equal to 1 for the current model settings:

$$\beta_{a,k}(t) = \frac{b \cdot c_{a,k}}{N(t)^{1-q}},$$

where $N(t)$ denotes the total population size at time t.

Thus, the force of infection (λ) for susceptible individual in age group a, at time t, was calculated as follows:

$$\lambda(a,t) = \left(1 + s_{amp} \cdot \exp\left(cos\left(\frac{2 \cdot \pi \cdot \left(t - s_{shift} \cdot 52\right)}{52}\right)\right)\right) \cdot \sum_k \left(\beta_{a,k} \cdot \frac{\sum_{i=1}^{4} \psi_i \cdot \left(A_i(k,t) + I_i(k,t)\right)}{N(k,t)}\right)$$

where s_{amp} is the seasonality amplitude, s_{shift} is the seasonal shift, $A_i(k,t)$ and $I_i(k,t)$ is the number of asymptomatic and symptomatic infectious individuals in age group k during their i-th infection at time t, ψ_i is the relative infectiousness of the i-th infection; and $N(k,t)$ is population size in age group k at time t.

S1.1.5 Ageing

The model accounted for ageing at weekly intervals. While all the individuals are undergoing an ageing process, only some of them transition to another age-defined compartment due to ageing. Thus, individuals who transition to another compartment due to ageing represent only a proportion of an age group, at the upper bound of age, who complete a full year of life over a given week. The ageing rate for the age group a was estimated as:

$$\xi(a) = \frac{1}{(a^{hight} - a^{low} + 1) \cdot 52},$$

where a^{low} and a^{hight} are the lower and upper bounds of an age group a, respectively, and 52 is the number of weeks in a year.

S1.1.6 Infection consequences

The modelling approach considered that RSV infection can be symptomatic (acute respiratory disease [ARD]), which can progress to lower respiratory disease (LRTD), LRTD can be associated with hospitalisation, and hospitalized LRTD cases can be fatal. The number of infection-related events (RSV infections, ARD, LRTD, hospitalisations and RSV-related deaths) was calculated outside the ODE, within the observational compartments INF, ARD, L, H and D, respectively, using the number of transitions from compartments S to E in the previous time step, and respective severity parameters.

The number of incident RSV infections (asymptomatic and symptomatic) was estimated using the number of transitions into the compartment E in the previous time point:

$$\begin{aligned} INF(a,t) &= \sum_{i=1}^{4} INF_i(a,t) + INF_V(a,t) = \sum_{i=1}^{4} INF_i(a,t) + \sum_{j=1}^{157} INF_V^j(a,t) \\ &= S_1(a,t-1) \cdot \lambda(a,t-1) + \sum_{i=2}^{4} S_i(a,t-1) \cdot \lambda(a,t-1) \cdot \sigma_{i-1} \\ &+ \sum_{j=1}^{157} V_4^j(a,t-1) \cdot \lambda(a,t-1) \cdot \sigma_3 \cdot \sigma_4^j, \end{aligned}$$

where $INF_i(a,t)$ is the number of RSV infections, which are i-th infection, for age group a, at time t, in unvaccinated individuals, $INF_V^j(a,t)$ is the number of RSV infections, for age group a, at time t, in vaccinated individuals in j-th week since vaccination, $S_i(a,t-1)$ is the number of individuals susceptible to RSV infection, for whom the potential infection will be the i-th previous infection, $\lambda(a,t-1)$ is a force of infection for age group a, at time $t-1$, σ_i is a relative risk of re-infection, after i-th infection, σ_4^j is a relative risk of RSV infection in vaccinated versus unvaccinated individuals, in j-th week since vaccination.

The number of ARD infections (*ARD*) was estimated using the number of incident RSV infections multiplied by the proportion of symptomatic infections among all infections.

$$\begin{aligned} ARD(a,t) &= \sum_{i=1}^{4} ARD_i(a,t) + ARD_V(a,t) = \sum_{i=1}^{4} ARD_i(a,t) + \sum_{j=1}^{157} ARD_V^j(a,t) \\ &= \sum_{i=1}^{4} INF_i(a,t) \cdot (1-p(a)) + \sum_{j=1}^{157} INF_V^j(a,t) \cdot (1-p(a)), \end{aligned}$$

where $ARD_i(a,t)$ is the number of ARD infections, which are i-th infection, for age group a, at time t, in unvaccinated individuals, $ARD_V^j(a,t)$ is the number of ARDSV infections, for age group a, at time t, in vaccinated individuals in j-th week since vaccination, σ_i is a relative risk of re-infection, after i-th infection, $p(a)$ is a proportion of infections that are asymptomatic, for age group a.

The number of LRTD infections (*L*) was estimated using the number of incident ARD, multiplied by the proportion of ARD infections that are LRTD. For protected individuals, this number was adjusted by the relative risk of LRTD given ARD in vaccinated versus unvaccinated individuals (σ_5^j).

$$L(a,t) = \sum_{i=1}^{4} L_i(a,t) + L_V(a,t) = \sum_{i=1}^{4} L_i(a,t) + \sum_{j=1}^{157} L_V^j(a,t) = \sum_{i=1}^{4} ARD_i(a,t) \cdot \kappa_i(a) + \sum_{j=1}^{157} ARD_V^j(a,t) \cdot \kappa_4(a) \cdot \sigma_5^j,$$

where $L_i(a,t)$ is the number of LRTD cases, which are i-th infection, for age group a, at time t, in unvaccinated individuals, $LRTD_V(a,t)$ is the number of LRTD cases, for age group a, at time t, in vaccinated individuals, $\kappa_i(a)$ is the proportion of ARD infections that are LRTDs, for age group a, for i-th infection, σ_5^j is a relative risk of LRTD given ARD in vaccinated versus unvaccinated individuals, in j-th week since vaccination.

The number of hospitalized cases (*H*) was estimated using the number of incident LRTD infections, multiplied by the proportion of hospitalisations due to LRTDs. For protected individuals, this number was adjusted by the relative risk of hospitalisation given LRTD in vaccinated versus unvaccinated individuals (σ_6^j).

$$H(a,t) = \sum_{i=1}^{4} H_i(a,t) + H_V(a,t) = \sum_{i=1}^{4} H_i(a,t) + \sum_{j=1}^{157} H_V^j(a,t) = \sum_{i=1}^{4} L_i(a,t) \cdot \delta_i(a) + \sum_{j=1}^{157} L_V^j(a,t) \cdot \delta_4(a) \cdot \sigma_6^j,$$

where $H_i(a,t)$ is the number of hospitalised LRTD cases, which are i-th infection, for age group a, at time t, in unvaccinated individuals, $H_V(a,t)$ is the number of hospitalized LRTD cases for age group a, at time t, in vaccinated individuals, $\delta_i(a)$ is a proportion of hospitalisations due to LRTD, for age group a, for i-th infection, σ_6^j is a relative risk of hospitalisation given LRTD in vaccinated versus unvaccinated individuals, in j-th week since vaccination.

The number of RSV-related deaths (*D*) was estimated using the number of incident hospitalized LRTD infections, multiplied by the proportion of deaths due to hospitalized LRTD.

$$D(a,t) = \sum_{i=1}^{4} D_i(a,t) + D_V(a,t) = \sum_{i=1}^{4} H_i(a,t) \cdot \mu(a) + H_V(a,t) \cdot \mu(a),$$

where $D_i(a,t)$ is the number of RSV-related deaths due to i-th infection, for age group a, at time t, $\mu(a)$ is the proportion of deaths due to hospitalized LRTD, for age group a.

S1.2 Model inputs

S1.2.1 Demographic inputs

Demographic calibration was informed by data on population size by age group for 1994 [1], 2023 [2], and, for exploratory analysis, 2030 [3] (**Table S3**).

Table S3. Population size reported in 1994 and 2023, and projected for 2030.

Age group	1994	2023	2030
0–4 years	3,852,159	3,572,007	3,460,590
5–9 years	3,806,147	3,925,921	3,569,885
10–14 years	3,633,584	4,150,287	3,925,960

Age group	1994	2023	2030
15–19 years	3,386,905	4,011,468	4,263,029
20–24 years	4,073,944	4,097,542	4,623,042
25–29 years	4,600,350	4,427,747	4,696,704
30–34 years	4,522,350	4,700,198	4,956,393
35–39 years	3,971,309	4,636,593	5,061,444
40–44 years	3,782,421	4,446,226	4,950,903
45–49 years	4,033,741	4,043,242	4,595,621
50–54 years	3,232,360	4,522,878	4,173,181
55–59 years	2,998,630	4,625,265	4,278,284
60–64 years	2,814,061	4,181,669	4,468,734
65–69 years	2,670,877	3,489,709	4,211,345
70–74 years	2,556,347	3,120,459	3,429,888
75–79 years	1,653,458	2,843,315	2,746,151
80–84 years	1,299,406	1,763,312	2,409,715
≥85 years	974,096	1,707,371	2,059,119
Total	57,862,145	68,265,209	71,879,988

Birth rates presented in **Table S4** were estimated from the data reported by the Office for National Statistics: the reported and projected number of births by year was divided by the total population size for the respective year [26].

Table S4. Birth rates, 1994–2054.

Year	Birth rate	Year	Birth rate	Year	Birth rate
1994	0.01297	2015	0.01194	2036	0.00948
1995	0.01261	2016	0.01180	2037	0.00955
1996	0.01261	2017	0.01143	2038	0.00962
1997	0.01246	2018	0.01101	2039	0.00970
1998	0.01226	2019	0.01067	2040	0.00977
1999	0.01193	2020	0.01016	2041	0.00983
2000	0.01153	2021	0.00998	2042	0.00988
2001	0.01132	2022	0.00979	2043	0.00991
2002	0.01127	2023	0.00972	2044	0.00993
2003	0.01166	2024	0.00975	2045	0.00992
2004	0.01194	2025	0.00973	2046	0.00989
2005	0.01196	2026	0.00967	2047	0.00984
2006	0.01231	2027	0.00961	2048	0.00978
2007	0.01259	2028	0.00956	2049	0.00970
2008	0.01285	2029	0.00951	2050	0.00962
2009	0.01269	2030	0.00948	2051	0.00953
2010	0.01286	2031	0.00945	2052	0.00943
2011	0.01276	2032	0.00944	2053	0.00934
2012	0.01276	2033	0.00943	2054	0.00925
2013	0.01215	2034	0.00943	-	-
2014	0.01202	2035	0.00944	-	-

Table S5 presents calibrated rate of population change by age group used in the model.

Table S5. Calibrated rate of population change, by age group.

Age group	Rate of population change, main analysis	Rate of population change, exploratory analysis (demographic shift)
0–4 years	0.000036	0.000072
5–9 years	-0.000197	-0.000004
10–14 years	-0.000063	-0.000251
15–19 years	-0.000076	-0.000208
20–24 years	-0.000048	-0.000281
25–29 years	-0.000324	-0.000118
30–34 years	-0.000323	-0.000300
35–39 years	0.000070	-0.000094
40–44 years	0.000063	-0.000027
45–49 years	0.000428	0.000212
50–54 years	-0.000496	0.000405
55–59 years	-0.000143	-0.000210
60–64 years	0.000273	-0.000275
65–69 years	0.000661	0.000067
70–74 years	0.000226	0.000828
75–79 years	0.000211	0.000769
80–84 years	0.002027	0.000316
≥85 years	0.003756	0.004337

S1.2.2 Contact matrix

The total number of physical and conversational contacts between different age groups by week was calculated using data from the POLYMOD study [7] and the R package socialmixr [8]. The UK population structure from the year of the POLYMOD study (2005) was utilized in the socialmixr package to ensure symmetry in the contact matrix. The matrix was constructed with the following assumptions:

- Symmetry of contacts (not symmetry of the matrix object).
- In the absence of age-stratified contact data for individuals aged 75 years and older, the contact pattern estimated for the ≥70 year-olds was applied uniformly across the 70–74, 75–79, 80–84, and ≥85-year age groups (i.e., equal by row, distributed by column).
- Social contacts data were weighted by the day of the week to consider different number of contacts in week days and weekends.
- Social contacts data were weighted based on the age distribution of the population.

The obtained contact matrix is presented in **Figure S1Error! Reference source not found.**.

Age group, y	0–4	5–9	10–14	15–19	20–24	25–29	30–34	35–39	40–44	45–49	50–54	55–59	60–64	65–69	70–74	75–79	80–84	85–100
0–4	13.1	5.8	3.3	2.1	3.7	5.8	6.2	7.9	3.1	2.1	2.5	2.2	1.5	0.7	0.5	0.4	0.3	0.3
5–9	5.6	46.6	8.4	4.2	3.8	5.1	7.4	10.8	7.8	2.7	2.4	1.8	2.3	1.9	0.9	0.7	0.5	0.4
10–14	2.9	7.8	49.3	9.0	1.8	2.6	4.0	7.8	8.5	5.2	3.0	2.5	1.4	1.4	1.1	0.9	0.7	0.6
15–19	1.8	3.8	8.7	45.5	8.1	5.0	3.3	6.2	6.5	6.3	4.1	2.2	1.4	2.1	1.6	1.3	1.0	0.8
20–24	3.3	3.4	1.8	8.2	18.2	9.4	6.0	5.2	5.4	7.4	4.2	3.3	2.3	1.8	1.1	0.9	0.7	0.5
25–29	5.4	4.8	2.6	5.3	9.8	12.5	7.9	6.4	6.2	6.4	6.0	5.1	3.4	2.8	0.8	0.7	0.5	0.4
30–34	5.1	6.4	3.7	3.1	5.6	7.1	11.3	8.9	7.0	5.3	4.5	4.6	3.2	1.8	0.8	0.7	0.5	0.4
35–39	5.8	8.2	6.4	5.2	4.3	5.1	7.9	10.6	8.6	6.2	4.6	3.9	3.9	3.4	1.0	0.8	0.6	0.5
40–44	2.3	6.0	7.0	5.5	4.5	5.0	6.3	8.7	9.6	8.5	5.0	4.1	3.7	2.6	2.0	1.7	1.3	1.0
45–49	1.8	2.4	5.0	6.3	7.2	6.1	5.5	7.3	9.9	13.2	5.6	5.0	3.8	2.0	1.7	1.4	1.1	0.9
50–54	2.4	2.3	3.2	4.5	4.5	6.2	5.1	6.0	6.4	6.2	5.5	7.0	3.2	2.1	2.3	1.9	1.5	1.2
55–59	1.9	1.6	2.4	2.3	3.3	4.9	4.9	4.7	4.9	5.1	6.5	9.0	5.8	3.0	1.8	1.5	1.1	0.9
60–64	1.7	2.6	1.7	1.7	2.9	4.1	4.3	5.8	5.5	4.9	3.7	7.3	4.6	4.6	2.1	1.8	1.3	1.1
65–69	1.0	2.4	1.9	3.1	2.7	3.9	2.8	5.9	4.5	3.0	2.8	4.4	5.3	5.3	2.9	2.4	1.8	1.4
70–74	0.8	1.3	1.8	2.7	1.8	1.3	1.5	1.9	4.0	2.9	3.6	2.9	2.8	3.3	3.4	2.8	2.2	1.7
75–79	0.8	1.3	1.8	2.7	1.8	1.3	1.5	1.9	4.0	2.9	3.6	2.9	2.8	3.3	3.4	2.8	2.2	1.7
80–84	0.8	1.3	1.8	2.7	1.8	1.3	1.5	1.9	4.0	2.9	3.6	2.9	2.8	3.3	3.4	2.8	2.2	1.7
85–100	0.8	1.3	1.8	2.7	1.8	1.3	1.5	1.9	4.0	2.9	3.6	2.9	2.8	3.3	3.4	2.8	2.2	1.7

Figure S1. Age-stratified weekly contact matrix (heatmap), with colour intensity indicating the average number of contacts between age groups.
y, years.

S1.2.3 Epidemiological inputs

The hospitalisation rate per 100,000 individuals was derived from national surveillance reports [9] and adjusted for underreporting [10] in age groups 0–64 years, while for individuals aged ≥65 years, the rate was based on estimates from Osei-Yeboah et al. [11] (**Table S6**).

Table S6. Hospitalisation rate, per 100,000 individuals (age group).

Age group	Calibration target	Source
0–4 years	609.544	National surveillance reports [30] adjusted for underreporting [10]
5–14 years	20.564	
15–44 years	7.764	
45–54 years	8.731	
55–64 years	21.780	
65–74 years	77.000	Osei-Yeboah et al. 2023 [11]
75–84 years	231.000	
≥85 years	395.000	

The weekly hospitalisation rate per 100,000 individuals was estimated by distributing the seasonal hospitalisation rate according to weekly hospitalisation data reported in national surveillance from week 17 of 2023 to week 16 of 2024 [9]. RSV-related hospitalisation rates per 100,000 individuals in the total population and in age groups used in demographic calibration are presented in **Table S7**.

Table S7. Weekly hospitalisation rate, per 100,000 individuals (by total population and age group), season 2023–2024, England.

Week	0–4 years	5–14 years	15–44 years	45–54 years	55–64 years	65–74 years	75–84 years	≥85 years	Total population
1	12.472	0.174	0.234	0.094	0.835	1.171	2.858	10.102	2.678
2	4.663	0.074	0.286	0.369	0.923	1.912	4.719	8.046	2.170
3	4.264	0.094	0.196	0.367	0.820	1.011	4.468	11.325	2.022
4	2.435	0.420	0.065	0.092	0.719	1.531	3.434	7.512	1.540

Week	0–4 years	5–14 years	15–44 years	45–54 years	55–64 years	65–74 years	75–84 years	≥85 years	Total population
5	2.051	0.085	0.033	0.092	0.102	1.138	1.064	5.932	0.869
6	2.860	0.194	0.067	0.095	0.000	0.132	0.223	1.130	0.517
7	2.635	0.090	0.242	0.196	0.109	0.542	0.000	0.000	0.641
8	1.386	0.164	0.063	0.000	0.000	0.122	0.205	2.079	0.366
9	0.640	0.094	0.033	0.094	0.209	0.519	0.871	1.638	0.437
10	0.640	0.189	0.065	0.092	0.000	0.126	0.638	0.539	0.295
11	2.051	0.085	0.065	0.000	0.000	0.126	0.213	1.079	0.379
12	1.373	0.081	0.000	0.000	0.099	0.240	0.403	2.500	0.385
13	0.677	0.092	0.000	0.103	0.000	0.000	0.000	1.816	0.198
14	0.657	0.097	0.000	0.000	0.105	0.000	0.000	1.124	0.164
15	1.052	0.000	0.067	0.000	0.000	0.000	0.660	0.000	0.230
16	1.085	0.000	0.000	0.000	0.108	0.000	0.000	0.586	0.165
17	0.455	0.000	0.000	0.000	0.000	0.000	0.000	0.000	0.049
18	0.417	0.000	0.000	0.000	0.000	0.122	0.000	0.509	0.090
19	0.981	0.111	0.000	0.000	0.000	0.000	0.000	0.000	0.139
20	0.638	0.000	0.000	0.000	0.000	0.000	0.000	0.728	0.095
21	0.366	0.000	0.000	0.000	0.000	0.000	0.000	0.000	0.040
22	0.000	0.000	0.000	0.000	0.000	0.000	0.000	0.000	0.184
23	0.829	0.000	0.000	0.000	0.000	0.000	0.000	0.000	0.091
24	0.776	0.000	0.000	0.000	0.000	0.000	0.000	0.000	0.084
25	0.000	0.143	0.000	0.000	0.000	0.000	0.000	0.000	0.040
26	0.000	0.000	0.000	0.000	0.000	0.000	0.000	0.000	0.184
27	0.340	0.000	0.107	0.000	0.000	0.000	0.000	0.000	0.110
28	0.829	0.000	0.000	0.000	0.000	0.000	0.000	0.000	0.091
29	0.766	0.000	0.000	0.000	0.000	0.000	0.000	0.000	0.084
30	0.802	0.000	0.088	0.000	0.000	0.170	0.000	0.000	0.178
31	1.946	0.000	0.000	0.000	0.000	0.000	0.000	0.736	0.245
32	0.802	0.111	0.044	0.000	0.000	0.000	0.000	0.000	0.148
33	1.258	0.000	0.000	0.000	0.155	0.000	0.310	0.000	0.206
34	0.774	0.000	0.000	0.000	0.000	0.225	0.000	0.000	0.119
35	2.048	0.000	0.000	0.000	0.000	0.000	0.000	0.000	0.226
36	1.268	0.111	0.040	0.000	0.000	0.000	0.000	0.000	0.193
37	1.755	0.000	0.048	0.000	0.000	0.000	0.311	0.000	0.259
38	3.901	0.000	0.076	0.000	0.000	0.000	0.000	0.000	0.453
39	7.915	0.000	0.000	0.000	0.131	0.000	0.272	0.698	0.923
40	6.121	0.093	0.032	0.091	0.000	0.378	0.213	0.545	0.834
41	12.550	0.310	0.072	0.000	0.000	0.000	0.473	0.607	1.531
42	18.769	0.501	0.035	0.000	0.000	0.000	0.913	0.584	2.254
43	26.995	1.345	0.144	0.000	0.114	0.281	0.473	1.822	3.489
44	32.194	0.660	0.262	0.092	0.307	0.632	1.702	3.775	4.316
45	33.473	1.131	0.360	0.367	0.615	0.632	1.489	3.775	4.748
46	31.965	1.162	0.609	0.665	1.477	2.212	3.518	7.303	5.696
47	39.017	1.131	0.131	0.643	1.639	1.517	4.468	10.247	6.224

Week	0–4 years	5–14 years	15–44 years	45–54 years	55–64 years	65–74 years	75–84 years	≥85 years	Total population
**48**	44.831	1.127	0.236	0.096	1.180	1.990	7.347	11.705	7.264
**49**	32.943	1.523	0.415	0.687	2.182	2.845	5.706	8.177	6.317
**50**	28.961	1.302	0.468	0.755	1.253	2.472	7.255	11.786	5.929
**51**	15.811	0.424	0.366	0.462	1.028	3.421	6.179	16.191	4.290
**52**	12.924	0.593	0.228	0.367	0.410	3.160	7.659	17.258	3.967

The initial values assumed for the proportion of ARD infections that progress to LRTD, based on available literature [12–14], are presented in **Table S8**. These values were adjusted during the epidemiological calibration process, and final calibrated values are presented in **Table S9**.

Table S8. Proportion of ARD infections that are LRTD – initial values used in calibration process.

Age group	Calibration target
0–4 years	27.0%
5–49 years	18.2%
50–64 years	28.4%
65–74 years	41.1%
≥75 years	52.1%

ARD, acute respiratory disease; LRTD, lower respiratory tract disease.

Age specific severity inputs used in the model are presented in **Table S9**. Other epidemiological parameters are presented in **Table S10**.

Table S9. Key age-stratified severity inputs.

	Parameter	Value	Source
$1-p$	Proportion of ARD among RSV infections*		
	0–4 years	84.3%	[15]
	5–14 years	47.9%	[15]
	15–39 years	23.7%	[15]
	40–54 years	22.2%	[15]
	55–64 years	29.7%	[16]
	65–74 years	44.3%	[16]
	≥75 years	76.3%	[16]
κ	Proportion of LRTD among ARD infections – main analysis		
	0–4 years	15.6%	Calibrated
	5–14 years	3.8%	Calibrated
	15–44 years	4.2%	Calibrated
	45–49 years	2.6%	Calibrated
	50–54 years	4.1%	Calibrated
	55–59 years	5.4%	Calibrated
	60–64 years	6.9%	Calibrated
	65–74 years	14.6%	Calibrated
	75–84 years	23.8%	Calibrated
	≥85 years	60.5%	Calibrated
κ	Proportion of LRTD among ARD infections – exploratory analysis		
	0–4 years	16.3%	Calibrated
	5–14 years	4.0%	Calibrated
	15–44 years	4.6%	Calibrated

	Parameter	Value	Source
	45–49 years	3.1%	Calibrated
	50–54 years	4.8%	Calibrated
	55–59 years	6.1%	Calibrated
	60–64 years	7.7%	Calibrated
	65–74 years	16.1%	Calibrated
	75–84 years	27.4%	Calibrated
	≥85 years	68.2%	Calibrated
δ	Proportion of hospitalisations among LRTD infections		
	0–4 years	10.8%	[17]
	5–49 years	2.1%	[10,12,13]
	50–64 years	5.9%	[10,12,13]
	65–74 years	10.1%	[10,12,13]
	≥75 years	18.7%	[10,12,13]
μ	Proportion of deaths among hospitalized cases		
	0–19 years	0.1%	[18]
	20–49 years	25.0%	[12,13]
	50–64 years	20.0%	[12,13]
	65–74 years	33.7%	[12,13]
	≥75 years	66.2%	[12,13]

* As the available sources reported the proportion of asymptomatic RSV infections, the proportion of ARD among all RSV infections was estimated as the complement, by subtracting the proportion of asymptomatic cases from 100%.

ARD, acute respiratory disease; LRTD, lower respiratory tract disease; RSV, respiratory syncytial virus.

Table S10. Key epidemiological parameters not stratified by age.

	Parameter	Value	Source
$1/\eta_m$	Duration of maternal immunity (days)	97	[19]
$1/\varepsilon$	Exposure to infection (Duration of latency period, days)	4.0035	[19]
σ	Relative risk of infection following previous, infections		
	1st infection	0.76	[20–23]
	2nd infection	0.60	
	3rd + infection	0.40	
$1/\eta_m$	Duration of infection (days)		
	1st infection	7.54	[19]
	2nd infection	6.52	[19]
	3rd + infection	4.04	[19]
$1/\eta$	Duration of antibody protection for those previously infected (days)	130	[19]
ψ	Relative infectiousness		
	2nd infection	0.75	[24]
	3rd + infection	0.51	[24]
b	Transmission probability – main analysis	0.08	Calibrated
	Transmission probability – exploratory analysis (demographic shift)	0.08	Calibrated
s	Seasonality parameters		
	Amplitude – main analysis	0.26	Calibrated
	Amplitude – exploratory analysis (demographic shift)	0.25	Calibrated
	Shift– main analysis	0.75	Calibrated
	Shift – exploratory analysis (demographic shift)	0.74	Calibrated

S1.3 Model calibration

S1.3.1 Calibration of demographic parameters

All-cause mortality and migration in the UK was calibrated to reproduce the population structure. Calibration was informed by the data on population size by age group, for a starting year (1994) and a target year (2023 for the main

analysis, 2030 for the exploratory analysis). For the demographic calibration process, the dynamic transmission model (DTM) was initialized with population size and distribution for 1994, within compartments M and S_1: it was assumed that 10% of age group 0–4 years is in M compartment and 90% of age group 0–4 years and all other age groups are in S_1 compartment. RSV infection was not included for demographic calibration.

The rate of population change by age group was calibrated to obtain age structure similar to that observed in a target year, using R function *optim*. The initial parameter values for optimization were defined in the code by the user. The number of individuals in each age group in a target year, as simulated by the model, was compared to the actual data (reported or projected for a target year), and the goodness of model fit was assessed using a likelihood function. The R function *optim* algorithm estimated new initial values for the next iteration and repeated the process until the maximum number of iterations was reached or a satisfactory result was achieved, as defined via maximization of a likelihood function.

Calibration of demographic parameters was conducted for the main analysis, and for an exploratory analysis on demographic shift.

S1.3.2 Calibration of epidemiological parameters

Three transmission parameters were calibrated to reproduce RSV incidence in population over time: (1) transmission probability from an infectious to a susceptible individual, (2) the amplitude of the seasonal cosine function to capture variations in infection intensity, and (3) the horizontal shift of the seasonal cosine function used to define infection seasonality at each week of the simulation. Calibration was performed using R function *optim*, in line with methodology described by Pitzer et al. [24]

The calibration target was set to the expected number of RSV-related hospitalisations, derived from Osei-Yeboah et al. [11], a reported hospitalisation rate [9] underreporting rate [10] and population size for each year of a calibration timeframe. In epidemiological calibration process the DTM was initialized by introducing a single infectious person, in a compartment I_1, in each age group. The RSV transmission was then simulated over the model time horizon.

Calibration timeframe, i.e., a time period used to compare simulated number of hospitalisations to the expected number of hospitalisations, was set to a 10-year period of 2023–2032.

Initial values for transmission probability, seasonal amplitude and seasonal shift were set to 0.1, 0.2, and 0.7, respectively.

In the first iteration, the model was run with the defined initial values over the period of 1994–2032. Then, a number of hospitalisations in each week in the calibration timeframe, as simulated by the model, was compared with the expected number of hospitalisations for 2023–2032, and the goodness of model fit was assessed using a likelihood function. The algorithm of R function *optim* estimated new initial values for the next iteration and repeated the process until the maximum number of iterations was reached or a satisfactory result was achieved, as defined via maximization of a likelihood function.

Calibration of epidemiologic parameters was performed using a step-wise procedure, guided by two main criteria: (1) fit of the simulated number of hospitalisations in the total population to the expected number of hospitalisations; (2) simulated age distribution of hospitalisations deviated from the expected age distribution. In case if the criterion on age distribution was not met, proportion of ARD infections that are LRTD was adjusted by a factor accounting for difference between simulated and expected number of hospitalisations, computed by age group. This model input was chosen for adjustment, as the previous literature review suggested that it is highly uncertain due to limited data availability. After this adjustment, model calibration was re-run, until a difference between simulated and actual number of hospitalisations in total population and in age group was acceptable. For this analysis, a ~5% deviation was considered as a target threshold.

Calibration of epidemiological parameters was conducted for the main analysis, and for an exploratory analysis on demographic shift.

S1.4 *Modelling of vaccination*

The model accounted for three types of vaccine protection specific to disease severity:

- Protection against RSV infection (symptomatic ARD or asymptomatic) – implemented as a relative risk of infection in vaccinated versus unvaccinated individuals, by time since vaccination (σ_4^j, where j is the number of weeks since vaccination);
- Protection against LRTD infection given ARD – implemented as a relative risk of LRTD given ARD, in vaccinated versus unvaccinated individuals, by time since vaccination (σ_5^j, where j is the number of weeks since vaccination);
- Protection against hospitalisation given LRTD – implemented as a relative risk of hospitalisation given LRTD, in vaccinated versus unvaccinated individuals, by time since vaccination (σ_6^j, where j is the number of weeks since vaccination).

Waning of post-vaccination immunity was explicitly modelled for each type of vaccine protection. The model allows consideration of weekly changes of the level of protection over 3 years (156 weeks) since vaccination, and a stable protection for the following period (157+ weeks).

Vaccine efficacy inputs were informed by a pivotal Phase 2/3 ConquerRSV trial for mRNA-1345, a case-driven, randomized, double-blind, placebo-controlled, multi-continent study, aiming to evaluate the safety and efficacy of the vaccine as compared with placebo in adults ≥ 60 years of age [25,26].

S1.4.1 Vaccine efficacy, main analysis

In the main analysis, a non-linear waning model was adopted to represent the time-dependent decline in vaccine efficacy. This approach is supported by accumulating evidence from multiple Phase 2/3 mRNA-1345 clinical studies, which indicate that protection provided by the vaccine does not decline at a constant rate. To accurately reflect this biologically plausible behavior, a non-linear function was incorporated into the model:

$$VE(t) = 1 - \exp\left(\beta_0 + \beta_1 \cdot \log(t)\right)$$

where $VE(t)$ is vaccine efficacy at time t in days, β_0 represents the initial log-odds of protection, β_1 captures the rate of decline in efficacy over time on the logarithmic scale.

Parameters of this function are provided in **Table S11**.

Estimated vaccine protection by week is presented in **Figure S2**.

Table S11. Vaccine efficacy inputs – non-linear waning

Parameter	Against ARD	Against LRTD
β_0	-1.9787039	-2.5107449
β_1	0.234412	0.3057026

ARD, acute respiratory disease; LRTD, lower respiratory tract disease.

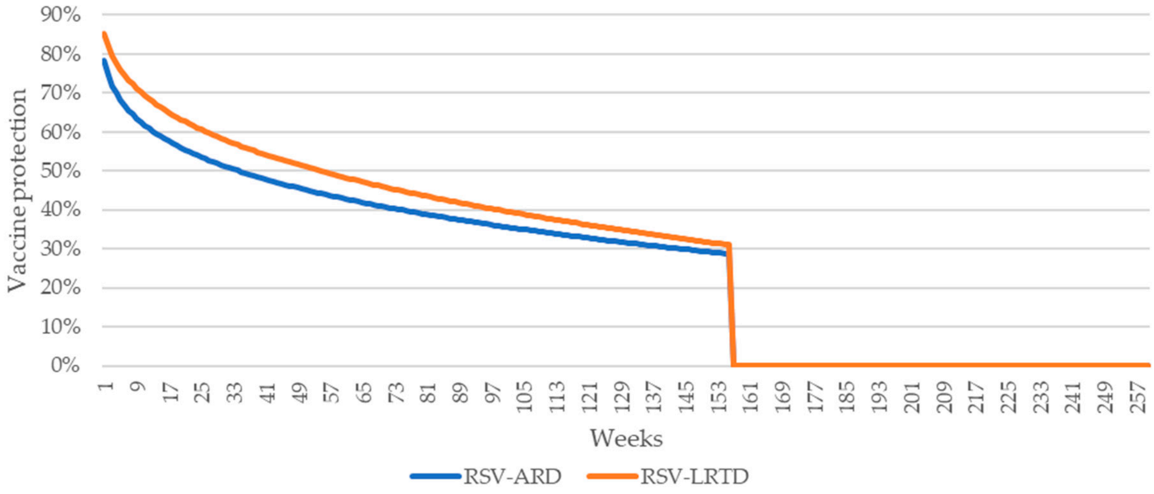

Figure S2. Vaccine protection by week since vaccination – main analysis

S1.4.2 Vaccine efficacy, exploratory analysis (alternative VE)

In the exploratory analysis, an alternative assumption on vaccine efficacy was tested, which included a third type of efficacy (against hospitalizations) and linear waning for all types of protection.

The initial vaccine efficacy at month 0 was set equal to the efficacy reported in the primary analysis of the ConquerRSV trial. Vaccine efficacy against RSV-LRTD with ≥2 symptoms was assessed at regular intervals over the follow-up period. A weighted least squares regression was performed on the vaccine efficacy estimates, using inverse variance weighting based on placebo case data. This approach produced monthly waning rates of 1.9% for RSV-LRTD and RSV hospitalisation, and 1.8% for RSV-ARD.

This waning rate was applied to linear decline of vaccine protection for the more severe endpoint of RSV-LRTD requiring inpatient care and the less severe endpoint of RSV-ARD.

Further, assumptions on initial vaccine protection and waning of protection were applied to derive inputs for the DTM, which required weekly estimates of vaccine efficacy, starting from week 1 post-vaccination. A linear waning function constructed as described above was used to compute vaccine efficacy function dependent on the number of weeks after vaccination.

Estimated vaccine protection by week is presented in **Figure S3**.

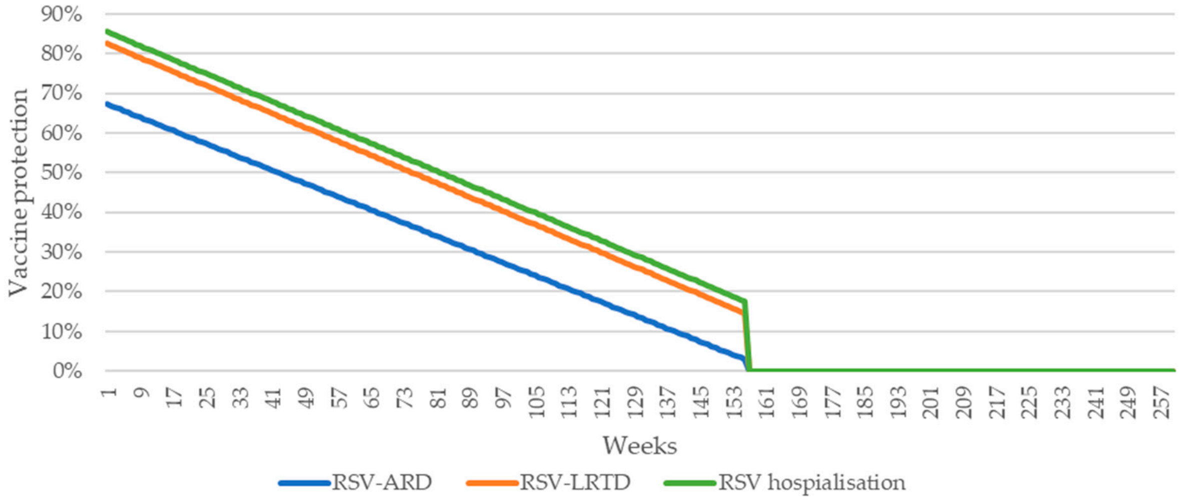

Figure S3. Vaccine protection by week since vaccination – exploratory analysis

S1.4.3 Vaccination coverage

An RSV vaccination coverage of 60% was assumed for individuals aged 65 years and older, consistent with observed RSV coverage measured as of 31 March 2025 [27]. Additionally, an alternative scenario tested uptake levels aligned with influenza vaccine coverage during the 2024–2025 season, estimated at approximately 80% [28].

To inform the weekly progression of RSV vaccination coverage in the model, cumulative influenza vaccination uptake data for individuals aged 65 years and older were used [29]. The number of influenza vaccine doses administered each week, from the start of the 2024–2025 influenza campaign through week 17, was expressed as a proportion of the total cumulative influenza coverage. These weekly proportions were then scaled to the 60% or 80% coverage target, under the assumption that RSV vaccination would follow a similar temporal pattern. Weekly RSV vaccination coverage inputs and corresponding influenza uptake data are presented in **Table S12**.

Table S12. Inputs for RSV vaccination coverage based on weekly influenza uptake in the 2024-2025 season.

Week since a start of vaccination programme	Cumulative number of influenza vaccinations administered	Proportion of final influenza vaccine uptake*	Estimated cumulative RSV vaccine coverage – influenza vaccine uptake	Estimated cumulative RSV vaccine coverage – influenza vaccine uptake
1	3,931,614	45.6%	27.4%	36.5%
2	5,453,986	63.3%	38.0%	50.7%
3	6,363,851	73.9%	44.3%	59.1%
4	7,374,237	85.6%	51.4%	68.5%
5	7,764,619	90.1%	54.1%	72.1%
6	8,019,298	93.1%	55.9%	74.5%
7	8,190,996	95.1%	57.1%	76.1%
8	8,316,351	96.5%	57.9%	77.2%
9	8,396,674	97.5%	58.5%	78.0%
10	8,480,678	98.5%	59.1%	78.8%
11	NA	NA	59.3%	79.0%**
12	NA	NA	59.4%	79.3%**
13	8,559,430	99.4%	59.6%	79.5%
14	8,583,279	99.6%	59.8%	79.7%
15	8,598,081	99.8%	59.9%	79.9%
16	8,608,673	99.9%	60.0%	80.0%
17	8,613,876	100.0%	60.0%	80.0%

*Estimated assuming that the cumulative number of influenza vaccinations administered by week 17 represents 100% of total uptake.

**Estimated by linear interpolation between weeks 10 and 13.

NA, data not available for weeks 11 and 12; RSV, respiratory syncytial virus.

S2 Supplement 2: Additional model results

S2.1 Model fit and calibration outputs

The model demonstrated a good fit to demographic and epidemiologic data considered for calibration.

Figures below (**Figure S4** to **Figure S7)** present results of demographic calibration. For the total population size, differences between the actual and simulated estimates ranged from 1% to 8% in the main analysis (population size slightly underestimated), and from −3% to 2% in the exploratory analysis on demographic shift (population size slightly overestimated for 2024 and underestimated for 2044). Simulated population size by age group closely corresponded to the target age distribution (as reported in 2023 for the main analysis or projected in 2030 for the exploratory analysis).

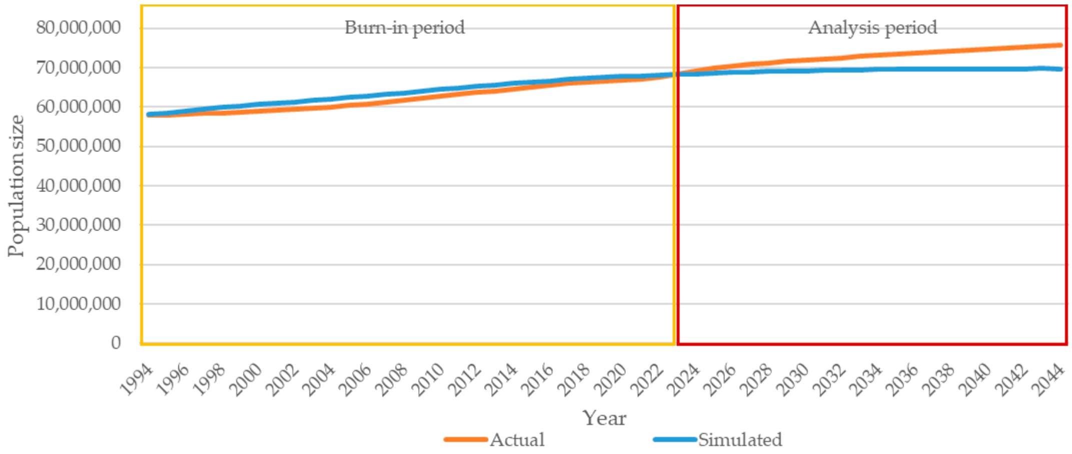

Figure S4. Total population size: comparison of model projections and target values over time (1994–2044) – main analysis. Actual data represent the reported population size, for 1994–2023 and projected population size for 2024–2044.

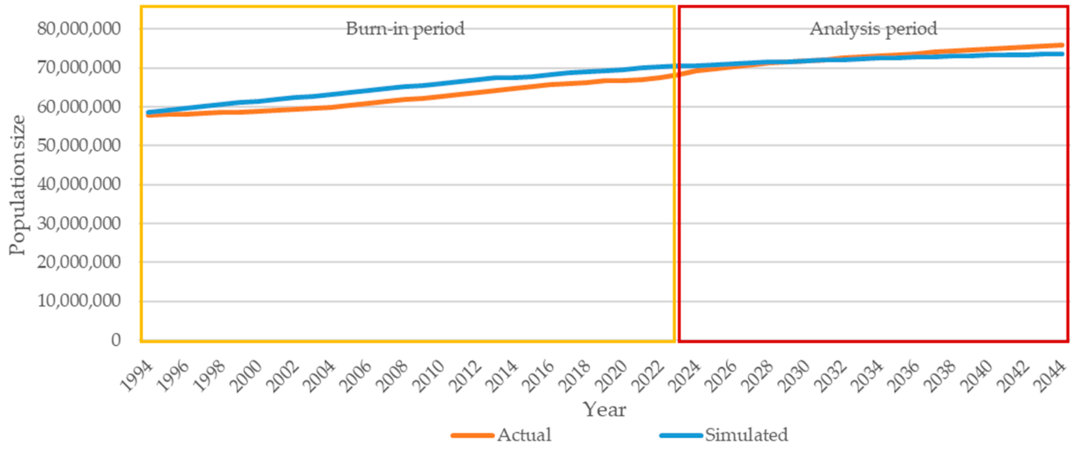

Figure S5. Total population size: comparison of model projections and target values over time (1994–2044) – exploratory analysis on demographic shift.
Actual data represent the reported population size for 1994–2023, and projected population size for 2024–2044.

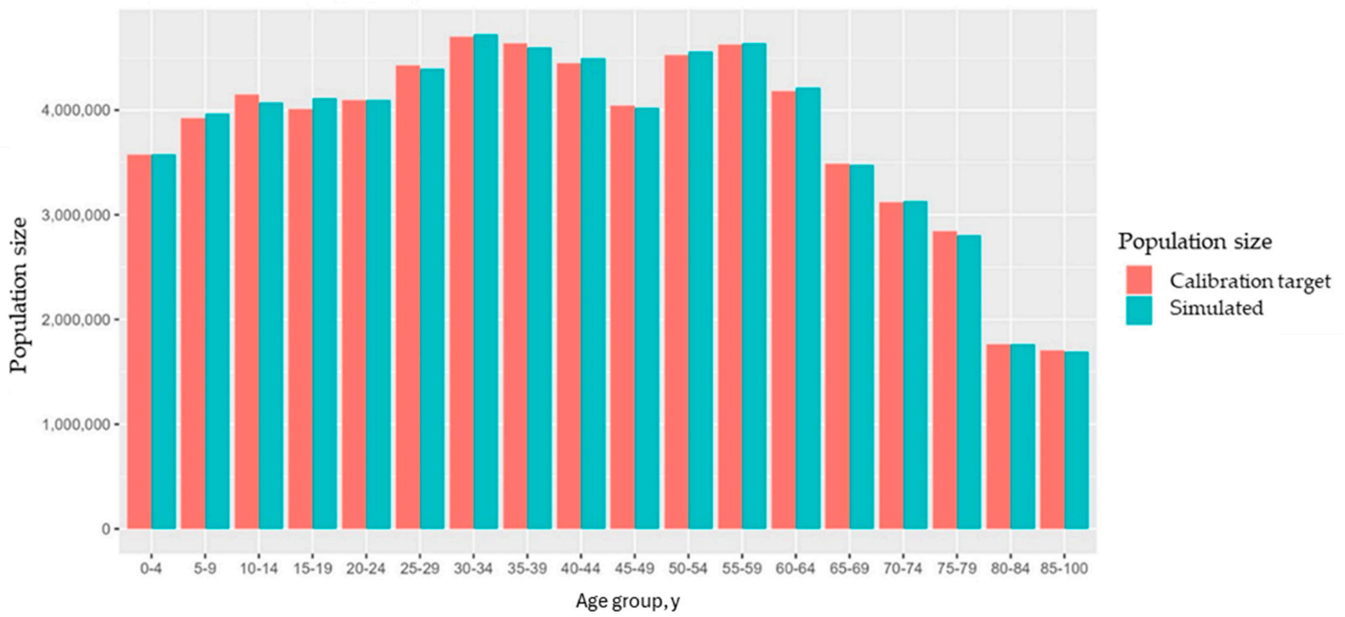

Figure S6. Population size by age group: comparison of model projections and calibration target values in 2023 – main analysis.
y, years.

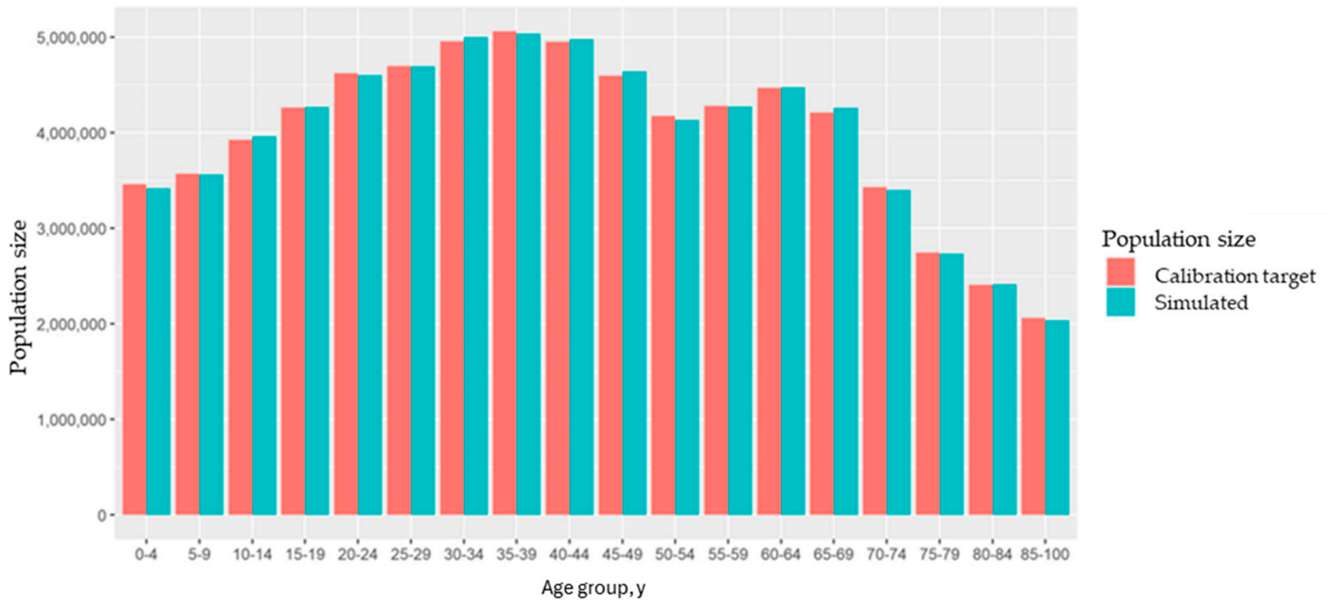

Figure S7. Population size by age group: comparison of model-simulated and calibration target values in 2030 – exploratory analysis on demographic shift.
y, years.

Prior to the start of the analytic time horizon, the model was run over a 30-year burn-in period (staring 1994), to allow for stabilization of RSV transmission dynamics. As shown in **Figure S8**, the designated analysis period corresponds to the phase in which stabilisation of the key epidemiological indicators was achieved.

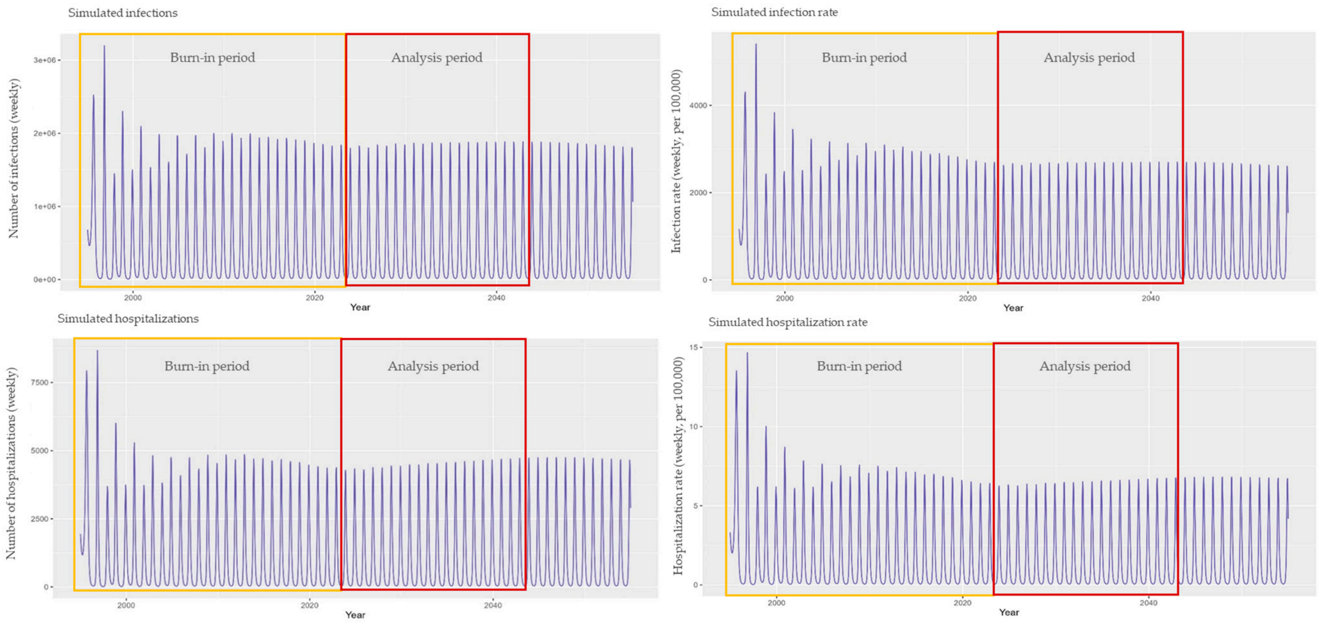

Figure S8. Simulated incidence of RSV infections and hospitalizations over the full model timeframe.

Figures and tables below (**Figure S9, Figure S10**, **Table S13** and **Table S14)**, present the results of epidemiologic calibration, with comparison between model projections and target hospitalization rates per 100,000 population, over the 2023–2032 calibration period. The model was calibrated to reproduce weekly hospitalisation rates in the total population, ensuring alignment with the observed seasonal patterns and allowing for the interannual variability. As shown in **Figure S9**, simulated weekly rates align closely with observed seasonal trends over the calibration period (2023–2032).

Total number of hospitalisations simulated by the model over 10 years was close to the expected actual number of hospitalisations, with a difference of 4%, which was considered acceptable (**Table S13).** Over the calibration timeframe, slight interannual variability was observed, with difference between the actual and simulated number of hospitalisations ranging from 2% to 6% across individual years.

Additionally, model inputs were refined to match age-specific hospitalisation patterns. The resulting average annual hospitalisation rates by age group, presented in **Figure S10** and **Table S14**, demonstrate strong concordance with target values.

These results support the reliability of the model's predictions of both the age distribution and temporal dynamics of RSV-related hospitalizations during the calibration period, providing a robust foundation for projections over the full analysis horizon, spanning from the 2024–2025 to 2043–2044 RSV seasons.

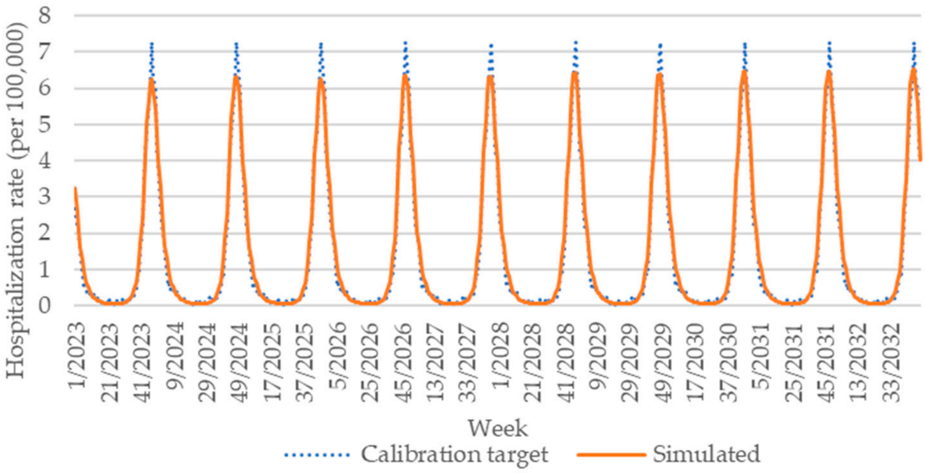

Figure S9. Weekly hospitalization rates per 100,000 population: comparison of model projections and calibration target values over time (2023–2032).

Table S13. Number of hospitalizations: comparison of model projections and calibration target values over time (2023–2032).

	**Overall**	**Year**									
	**2023–2032**	**2023**	**2024**	**2025**	**2026**	**2027**	**2028**	**2029**	**2030**	**2031**	**2032**
Simulated	531,390	51,628	52,812	51,872	53,227	52,585	53,708	53,213	54,113	53,752	54,479
Actual	511,120	50,693	50,803	50,906	51,004	51,096	51,181	51,259	51,331	51,395	51,452
Difference	4%	2%	4%	2%	4%	3%	5%	4%	5%	5%	6%

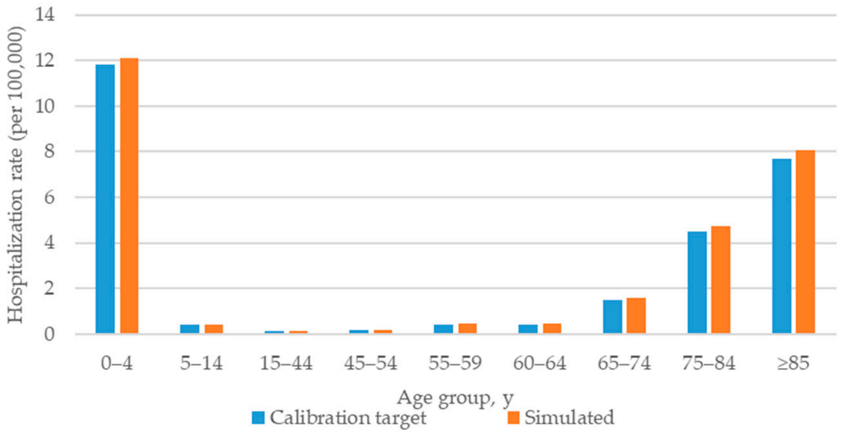

Figure S10. Comparison of model projections and calibration target hospitalization rates by age group (per 100,000 population), based on the average annual rate over the 2023–2032 calibration period.
y, years.

Table S14. Total number of hospitalisations (2023–2032): comparison of model projections and calibration target values by age group.

	Overall	Age group, years								
	2023–2032	0–4	5–14	15–44	45–54	55–59	60–64	65–74	75–84	≥85
Simulated	531,390	214,862	16,509	21,708	7,974	10,844	9,929	55,914	118,212	75,439
Actual	511,120	209,522	16,094	20,890	7,627	10,352	9,475	53,228	112,292	71,639
Difference	4%	3%	3%	4%	5%	5%	5%	5%	5%	5%

S2.2 Additional validation results

Comparison of model projections and estimates reported in the literature for age-specific annual incidence rates of ARD per 100,000 individuals is presented in **Table S15**. Published values vary substantially across age groups, reflecting heterogeneity in the available data. Previous studies have highlighted inconsistencies in case definitions, data collection methods, and population sampling, which contribute to the observed variability in reported incidence rates. Despite these differences, the ARD incidence rates predicted by the DTM fall within or remain comparable to the reported ranges, indicating broad consistency between model outputs and existing evidence.

Table S15. ARD rate per 100,000 in model prediction and available literature.

Age group, years	DTM outputs	Literature	Source
16+	7,419	1,600–7,400	Wilkinson 2023 et al. [30]
18+	7,321	900–13,100	Wilkinson 2023 et al. [30]
18+	7,321	6,481	RAND [31]
15–24	9,295	10,300	Wilkinson 2023 et al. [30]
15–44	9,186	~2,000–6,010	Wilkinson 2023 et al. [30]
25–44	9,137	10,200	Wilkinson 2023 et al. [30]
18–49	8,792	6,252	RAND [31]
45+	5,970	6,120–7,620	Wilkinson 2023 et al. [30]
45–64	6,465	17,900	Wilkinson 2023 et al. [30]
50–64	6,198	7,073	RAND [31]
60+	5,385	4,570–5,750	Wilkinson 2023 et al. [30]
65–74	5,536	7,217	RAND [31]
75+	5,059	6,208	RAND [31]

DTM outputs are presented for a season 2024/2025, for a strategy without vaccination.

ARD rate based on RAND was estimated dividing the reported number of cases by average population size reported for 2024/2025 RSV season.

ARD, acute respiratory disease; DTM, dynamic transmission model.

Comparison of model projections and estimates reported in the literature for age-specific annual hospitalisation rates per 100,000 individuals, is presented in **Table S16**. Despite variability across data sources, the hospitalisation rates predicted by the DTM were within the reported ranges, for the majority of age groups, indicating broad consistency between model outputs and available empirical evidence. Description of validation presented in the main text is focused on key independent sources. **Table S16** also includes additionally the data from United Kingdom Health Security Agency (UKHSA) upscaled by 1.5 and Osei-Yeboah et al., which were used as a calibration target, and therefore present a limited value for the model validation [32].

Table S16. Hospitalisation rate per 100,000 in model prediction and available literature.

Age group, years	DTM outputs	UKHSA data [9]	UKHSA data [9], upscaled by 1.5 [10]	Osei-Yeboah et al. [11]	Howa et al. [33]	RAND [31]	Fleming et al. [12]	Sharp et al. [34]	Hodgson et al. [35]	Johannesen et al. [32]	Zhang et al. [36]
0–4	608	406	610	NA	NA	NA	NA	NA	NA	NA	NA
5–9	23	14	21	NA	NA	NA	NA	NA	4	NA	NA
10—14	18			NA	NA	NA	NA	NA		NA	NA
15—19	9	5	8	NA	NA	NA	NA	NA	30	NA	NA
20—24	7			7*	8*	4*	3–5	NA		10	NA
25—29	7							NA			NA
30—34	8							NA			NA
35—39	9							NA			NA
40–44	8							NA			NA
45–49	4	6	9					NA			NA
50–54	13				32	30	22–36	NA			NA
55–59	22	15	22					NA			NA
60–64								NA			105
65–69	82	29	43	77	91	86	62–101	71	71	90	128
70–74											185
75–79	237	68	102	231		234	180–291	251	251	280	227
80–84											
85–100	405	152	228	395						600	752
**≥ 65**	**178**	**59**	**88**	**174**	**91**	**158**	**119-193**	**159**	**159**	**225**	**276**
**Total**	**75**	**32**	**47**	**NA**	**NA**	**NA**	**NA**	**NA**	**NA**	**NA**	**NA**

*For the RAND, Howa et al., and Osei-Yeboah et al., hospitalisation rates were originally reported for populations aged 18 years and older. In this analysis, values are presented starting from 20 years of age to match the age stratification used in the model.
DTM outputs are presented for the 2024–2025, for a strategy without vaccination.
Hospitalisation rate for individuals aged 65 years and older was estimated using data reported in the literature and simulated population size in this age group at the end of 2024–2025 season (week 9 of 2025).
DTM, dynamic transmission model; NA, not available; UKHSA, UK Health Security Agency.

S2.3 RSV vaccination strategies: impact in target and total population

The number vaccine doses administered, number of cases, number of cases avoided, and percentage of cases avoided with Strategies 5-8, in the target and total population are presented in **Table S17** and **Table S18,** respectively.

Table S17. Number of cases, number of cases avoided, and percentage of cases avoided in the **target population**, mRNA-1345 vs no vaccination over 20 years.

**Strategy**	**No vaccination**		**Strategy 5**	**Strategy 6**	**Strategy 7**	**Strategy 8**
**Age group**	**75–80 y**	**⩾ 60 y**	**⩾ 60 y**	**75–80 y**	**75–80 y**	**⩾ 60 y**
**Coverage**	**-**	**-**	**60%**	**80%**	**60%**	**80%**
**Revaccination**	**-**	**-**	**No**	**No**	**Every 3 y**	**Every 3 y**
Number of vaccine doses						
Number of vaccine doses administered	-	-	19,906,760	11,902,883	27,249,055	106,397,904
Number of cases						
RSV ARD	4,195,188	20,053,473	18,358,175	3,450,965	3,164,318	12,499,634

Strategy	No vaccination		Strategy 5	Strategy 6	Strategy 7	Strategy 8
RSV LRTD	997,987	3,725,497	3,444,496	800,336	725,173	2,113,360
RSV hospitalisation	186,227	552,758	514,920	149,345	135,319	307,534
RSV death	123,356	318,698	298,031	98,925	89,635	175,004
Number of cases avoided						
RSV ARD	-	-	1,695,298	744,224	1,030,870	7,553,838
RSV LRTD	-	-	281,001	197,651	272,814	1,612,137
RSV hospitalisation	-	-	37,838	36,882	50,908	245,225
RSV death	-	-	20,667	24,431	33,721	143,694
Percentage of cases avoided						
RSV ARD	-	-	8%	18%	25%	38%
RSV LRTD	-	-	8%	20%	27%	43%
RSV hospitalisation	-	-	7%	20%	27%	44%
RSV death	-	-	6%	20%	27%	45%

Percentages of cases avoided are calculated using the total number of cases without vaccination as a denominator, for each respective target age group. Due to variation in population size, percentages are not directly comparable between strategies with different eligibility criteria.

ARD, acute respiratory disease; LRTD, lower respiratory tract disease; y, years; RSV, respiratory syncytial virus; y, years.

Table S18. Number of cases, number of cases avoided, and percentage of cases avoided in the **total population**, mRNA-1345 vs no vaccination, over 20 years

Strategy	No vaccination	Strategy 5	Strategy 6	Strategy 7	Strategy 8
**Age group**	**Total population**	**≥ 60 y**	**75–80 y**	**75–80 y**	**≥ 60 y**
**Coverage**	**-**	**60%**	**80%**	**60%**	**80%**
**Revaccination**	**-**	**No**	**No**	**Every 3 y**	**Every 3 y**
Number of vaccine doses					
Number of vaccine doses administered	-	19,906,760	11,902,883	27,249,055	106,397,904
Number of cases					
RSV ARD	153,877,864	151,034,407	152,558,805	151,307,205	142,031,599
RSV LRTD	12,025,486	11,681,296	11,752,142	11,430,074	10,178,790
RSV hospitalisation	1,090,248	1,048,844	1,042,852	986,686	831,911
RSV death	336,077	315,171	305,694	269,598	191,499
Number of cases avoided					
RSV ARD	-	2,843,456	1,319,059	2,570,659	11,846,264
RSV LRTD	-	344,189	273,344	595,411	1,846,696
RSV hospitalisation	-	41,403	47,396	103,562	258,336
RSV death	-	20,906	30,384	66,479	144,578
Percentage of cases avoided					
RSV ARD	-	2%	1%	2%	8%
RSV LRTD	-	3%	2%	5%	15%
RSV hospitalisation	-	4%	4%	9%	24%
RSV death	-	6%	9%	20%	43%

ARD, acute respiratory disease; LRTD, lower respiratory tract disease; RSV, respiratory syncytial virus; y, years.

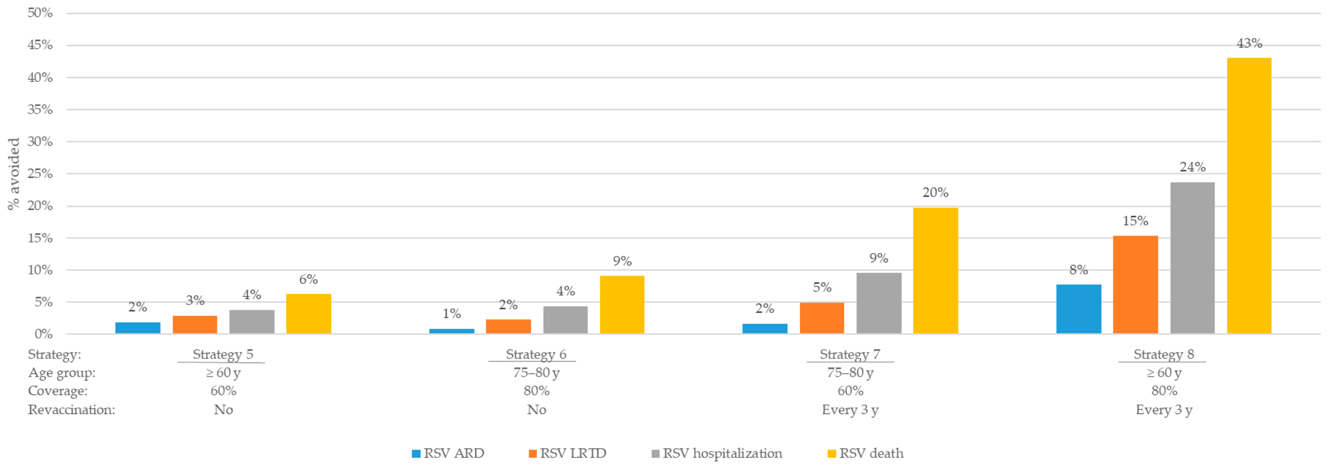

Figure S11. Percentage of cases avoided in **total population**, mRNA-1345 vs no vaccination (over 20 years). ARD, Acute respiratory disease; LRTD, Lower respiratory tract disease; RSV, Respiratory syncytial virus; y, years.

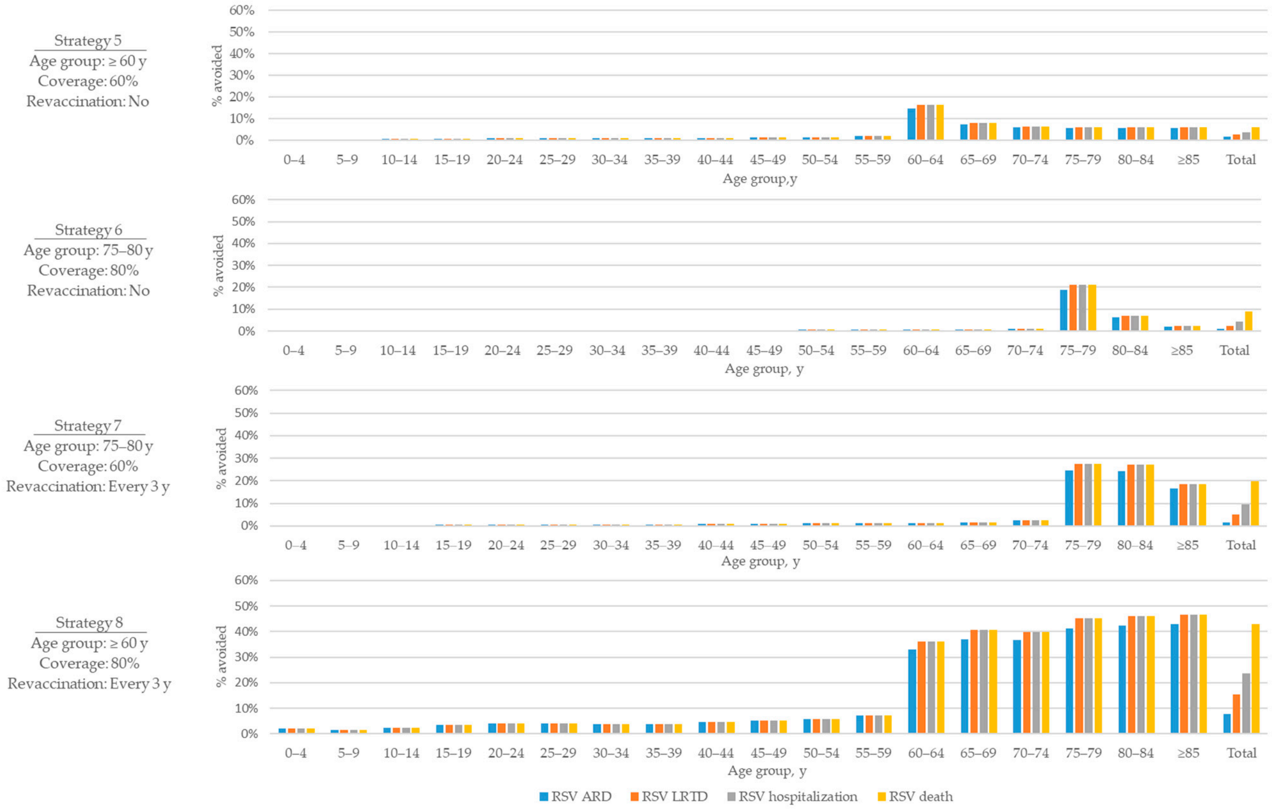

Figure S12. Percentage of cases avoided in **total population** by age group, mRNA-1345 vs no vaccination (over 20 years). ARD, Acute respiratory disease; LRTD, Lower respiratory tract disease; RSV, Respiratory syncytial virus; y, years.

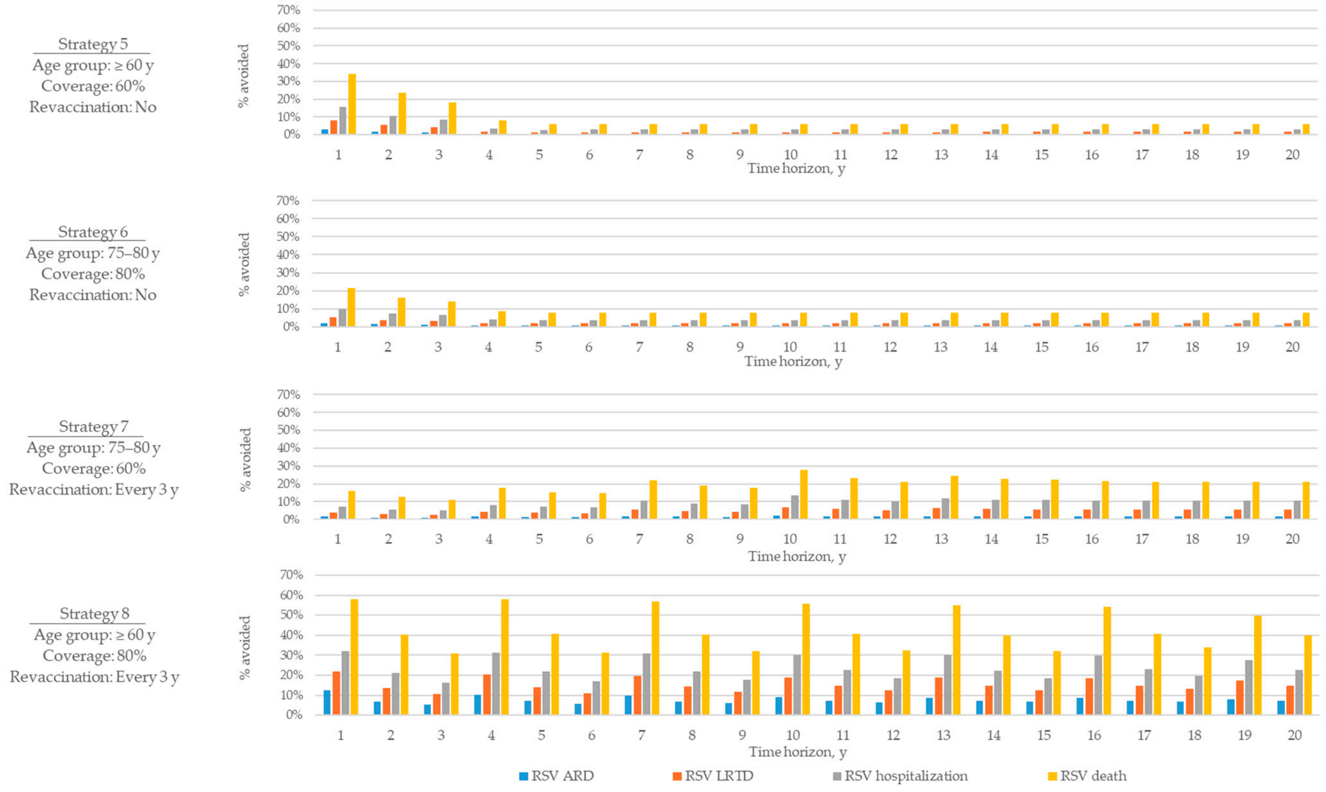

Figure S13. Percentage of cases avoided in **total population** over time, mRNA-1345 vs no vaccination.
ARD, Acute respiratory disease; LRTD, Lower respiratory tract disease; RSV, Respiratory syncytial virus; y, years.

S2.4 Additional results of the exploratory analysis

Exploratory analysis was conducted for Strategies 4 and 8 only. The observed trends in disease impact were similar for these two strategies, therefore the results for Strategy 4 are presented in the main text, and corresponding outputs for Strategy 8 are provided below (**Table S19**).

Table S19. Exploratory analysis: Number of cases, number of cases avoided, and percentage of cases avoided in the **total population**, mRNA-1345 vs no vaccination, over 20 years (Strategy 8)

**Strategy**	**No vaccination**	**Strategy 8**	**Strategy 8**	**No vaccination**	**Strategy 8**
**Analysis**	**Main analysis**	**Main analysis**	**Exploratory analysis, alternative VE**	**Exploratory analysis, demographic shift**	**Exploratory analysis, demographic shift**
			Number of vaccine doses		
Number of vaccine doses administered	-	106,397,904	106,397,904	-	114,182,629
			Number of cases		
RSV ARD	153,877,864	142,031,599	142,971,964	146,958,230	135,794,816
RSV LRTD	12,025,486	10,178,790	10,123,650	12,523,099	10,555,908
RSV hospitalisation	1,090,248	831,911	811,197	1,141,781	866,035
RSV death	336,077	191,499	178,969	358,961	204,501
			Number of cases avoided		
RSV ARD	-	11,846,264	10,905,900	-	11,163,414
RSV LRTD	-	1,846,696	1,901,835	-	1,967,191
RSV hospitalisation	-	258,336	279,050	-	275,746
RSV death	-	144,578	157,108	-	154,461

Strategy	No vaccination	Strategy 8	Strategy 8	No vaccination	Strategy 8
**Analysis**	**Main analysis**	**Main analysis**	**Exploratory analysis, alternative VE**	**Exploratory analysis, demographic shift**	**Exploratory analysis, demographic shift**
			Proportion of cases avoided		
RSV ARD	-	8%	7%	-	8%
RSV LRTD	-	15%	16%	-	16%
RSV hospitalisation	-	24%	26%	-	24%
RSV death	-	43%	47%	-	43%

ARD, acute respiratory disease; LRTD, lower respiratory tract disease; RSV, respiratory syncytial virus; VE, vaccine efficacy.

It should be noted that in the exploratory analysis on a demographic shift, a more pronounced population growth was projected in comparison to the main analysis. While in the main analysis population size increased by around 1.2 million over 20 years, in the exploratory analysis a 2.9 million increase over the same time period was simulated (see **Figure S14**). Additionally, this exploratory analysis simulated change in the age distribution, with increasing proportion of the population in the older age groups. Particularly, over 2024–2044 years, the population size in the age group of ≥60 year olds increased by 2.1 million in the main analysis, and by 2.7 million in the exploratory analysis (see **Figure S15**).

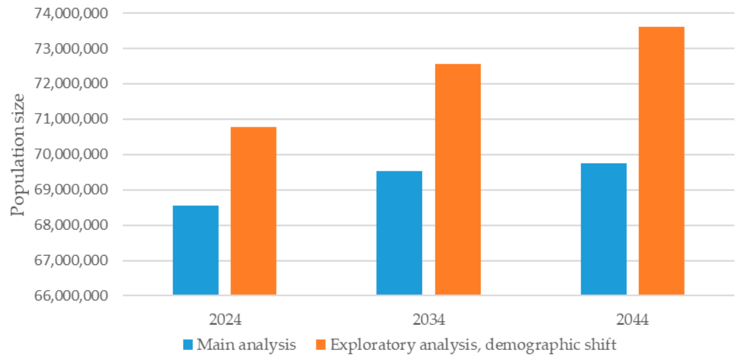

Figure S14. Projected population size for main analysis and exploratory analysis in 2024, 2034, and 2044.

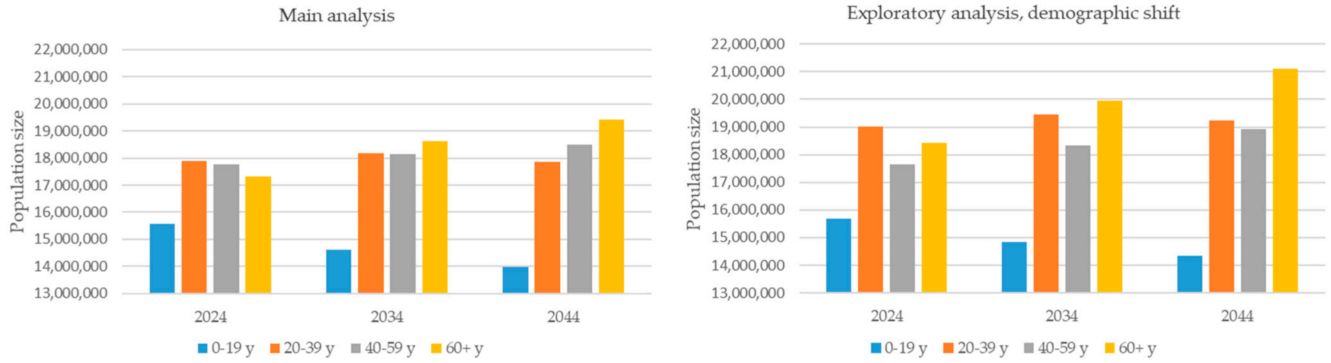

Figure S15. Projected population structure by age for main analysis and exploratory analysis in 2024, 2034, and 2044.
y, years.

References

1. Office for National Statistics. UK Population Estimates 1851 to 2014. . Available online: https://www.ons.gov.uk/peoplepopulationandcommunity/populationandmigration/populationestimates/adhocs/004356ukpopulationestimates1851to2014 (accessed on 29 January 2025).

2. Office for National Statistics. Mid-year population estimates, United Kingdom, June 2023. Available online: https://www.ons.gov.uk/file?uri=/peoplepopulationandcommunity/populationandmigration/populationestimates/datasets/populationestimatesforukenglandandwalesscotlandandnorthernireland/mid2023/mye23tablesuk.xlsx (accessed on 29 January 2025).
3. Office for National Statistics. National population projections: 2022-based. Available online: https://www.ons.gov.uk/file?uri=/peoplepopulationandcommunity/populationandmigration/populationprojections/datasets/tablea11principalprojectionuksummary/2022based/ukpppsummary.xlsx (accessed on 29 January 2025).
4. Office for National Statistics. Vital statistics in the UK: births, deaths and marriages. Available online: (accessed on 29 January 2025).
5. Office for National Statistics. National population projections: 2020-based. Year ending June 2022 estimated international migration variant. Available online: https://www.ons.gov.uk/file?uri=/peoplepopulationandcommunity/populationandmigration/populationprojections/datasets/2020basedinterimnationalpopulationprojectionsyearendingjune2022estimatedinternationalmigrationvariant/uksummary/ukppvsummary20.xls (accessed on 29 January 2025).
6. Office for National Statistics. Population estimates for the UK and constituent countries by sex and age; historical time series. Available online: https://www.ons.gov.uk/file?uri=/peoplepopulationandcommunity/populationandmigration/populationestimates/datasets/populationestimatesforukenglandandwalesscotlandandnorthernireland/ukpopulationestimates1838to2023/ukpopulationestimates183820231.xlsx (accessed on 29 January 2025).
7. Mossong, J.; Hens, N.; Jit, M.; Beutels, P.; Auranen, K.; Mikolajczyk, R.; Massari, M.; Salmaso, S.; Tomba, G.S.; Wallinga, J.; et al. Social contacts and mixing patterns relevant to the spread of infectious diseases. *PLoS Med* **2008**, *5*, e74, doi:10.1371/journal.pmed.0050074.
8. Funk, S. Introduction to socialmixr. Available online: https://cran.r-project.org/web/packages/socialmixr/vignettes/socialmixr.html (accessed on 29 January 2025).
9. UK Health Security Agency. National flu and COVID-19 surveillance reports: 2 May 2024 (week 18). Available online: https://www.gov.uk/government/statistics/national-flu-and-covid-19-surveillance-reports-2023-to-2024-season (accessed on 12 June 2024).
10. McLaughlin, J.M.; Khan, F.; Begier, E.; Swerdlow, D.L.; Jodar, L.; Falsey, A.R. Rates of medically attended RSV among US adults: a systematic review and meta-analysis. *Open Forum Infect Dis* **2022**, *9*, ofac300, doi:10.1093/ofid/ofac300.
11. Osei-Yeboah, R.; Spreeuwenberg, P.; Del Riccio, M.; Fischer, T.K.; Egeskov-Cavling, A.M.; Bøås, H.; van Boven, M.; Wang, X.; Lehtonen, T.; Bangert, M.; et al. Estimation of the number of respiratory syncytial virus-associated hospitalizations in adults in the European Union. *J Infect Dis* **2023**, *228*, 1539-1548, doi:10.1093/infdis/jiad189.
12. Fleming, D.M.; Taylor, R.J.; Lustig, R.L.; Schuck-Paim, C.; Haguinet, F.; Webb, D.J.; Logie, J.; Matias, G.; Taylor, S. Modelling estimates of the burden of respiratory syncytial virus infection in adults and the elderly in the United Kingdom. *BMC Infect Dis* **2015**, *15*, 443, doi:10.1186/s12879-015-1218-z.
13. Korsten, K.; Adriaenssens, N.; Coenen, S.; Butler, C.; Ravanfar, B.; Rutter, H.; Allen, J.; Falsey, A.; Pircon, J.Y.; Gruselle, O.; et al. Burden of respiratory syncytial virus infection in community-dwelling older adults in Europe (RESCEU): an international prospective cohort study. *Eur Respir J* **2021**, *57*, doi:10.1183/13993003.02688-2020.
14. Ohuma, E.O.; Okiro, E.A.; Ochola, R.; Sande, C.J.; Cane, P.A.; Medley, G.F.; Bottomley, C.; Nokes, D.J. The natural history of respiratory syncytial virus in a birth cohort: the influence of age and previous infection on reinfection and disease. *Am J Epidemiol* **2012**, *176*, 794-802, doi:10.1093/aje/kws257.

15. Munywoki, P.K.; Koech, D.C.; Agoti, C.N.; Bett, A.; Cane, P.A.; Medley, G.F.; Nokes, D.J. Frequent asymptomatic respiratory syncytial virus infections during an epidemic in a rural Kenyan household cohort. *J Infect Dis* **2015**, *212*, 1711-1718, doi:10.1093/infdis/jiv263.
16. Krauer, F.; Guenther, F.; Treskova-Schwarzbach, M.; Schoenfeld, V.; Koltai, M.; Jit, M.; Hodgson, D.; Schneider, U.; Wichmann, O.; Harder, T.; et al. Effectiveness and efficiency of immunisation strategies to prevent RSV among infants and older adults in Germany: a modelling study. *BMC Med* **2024**, *22*, 478, doi:10.1186/s12916-024-03687-3.
17. Li, Y.; Wang, X.; Blau, D.M.; Caballero, M.T.; Feikin, D.R.; Gill, C.J.; Madhi, S.A.; Omer, S.B.; Simoes, E.A.F.; Campbell, H.; et al. Global, regional, and national disease burden estimates of acute lower respiratory infections due to respiratory syncytial virus in children younger than 5 years in 2019: a systematic analysis. *Lancet* **2022**, *399*, 2047-2064, doi:10.1016/S0140-6736(22)00478-0.
18. Cromer, D.; van Hoek, A.J.; Newall, A.T.; Pollard, A.J.; Jit, M. Burden of paediatric respiratory syncytial virus disease and potential effect of different immunisation strategies: a modelling and cost-effectiveness analysis for England. *Lancet Public Health* **2017**, *2*, e367-e374, doi:10.1016/s2468-2667(17)30103-2.
19. Hodgson, D.; Pebody, R.; Panovska-Griffiths, J.; Baguelin, M.; Atkins, K.E. Evaluating the next generation of RSV intervention strategies: a mathematical modelling study and cost-effectiveness analysis. *BMC Med* **2020**, *18*, 348, doi:10.1186/s12916-020-01802-8.
20. Glezen, W.P.; Taber, L.H.; Frank, A.L.; Kasel, J.A. Risk of primary infection and reinfection with respiratory syncytial virus. *Am J Dis Child* **1986**, *140*, 543-546, doi:10.1001/archpedi.1986.02140200053026.
21. Hall, C.B.; Geiman, J.M.; Biggar, R.; Kotok, D.I.; Hogan, P.M.; Douglas, G.R., Jr. Respiratory syncytial virus infections within families. *N Engl J Med* **1976**, *294*, 414-419, doi:10.1056/nejm197602192940803.
22. Henderson, F.W.; Collier, A.M.; Clyde, W.A., Jr.; Denny, F.W. Respiratory-syncytial-virus infections, reinfections and immunity. A prospective, longitudinal study in young children. *N Engl J Med* **1979**, *300*, 530-534, doi:10.1056/nejm197903083001004.
23. Monto, A.S.; Lim, S.K. The Tecumseh study of respiratory illness. 3. Incidence and periodicity of respiratory syncytial virus and *Mycoplasma pneumoniae* infections. *Am J Epidemiol* **1971**, *94*, 290-301, doi:10.1093/oxfordjournals.aje.a121322.
24. Pitzer, V.E.; Viboud, C.; Alonso, W.J.; Wilcox, T.; Metcalf, C.J.; Steiner, C.A.; Haynes, A.K.; Grenfell, B.T. Environmental drivers of the spatiotemporal dynamics of respiratory syncytial virus in the United States. *PLoS Pathog* **2015**, *11*, e1004591, doi:10.1371/journal.ppat.1004591.
25. ModernaTX Inc. Final cost-effectiveness of Moderna's RSV vaccine, mRESVIA (mRNA-1345) compared to no vaccination in adults ≥60 years of age in the United States. Data on file. **2024**.
26. Wilson, E.; Goswami, J.; Baqui, A.H.; Doreski, P.A.; Perez-Marc, G.; Zaman, K.; Monroy, J.; Duncan, C.J.A.; Ujiie, M.; Rämet, M.; et al. Efficacy and safety of an mRNA-based RSV preF vaccine in older adults. *N Engl J Med* **2023**, *389*, 2233-2244, doi:10.1056/NEJMoa2307079.
27. UK Health Security Agency. RSV vaccine coverage report in older adults for catch-up cohorts in England: March 2025. Available online: https://www.gov.uk/government/publications/rsv-immunisation-for-older-adults-and-pregnant-women-vaccine-coverage-in-england/rsv-vaccine-coverage-report-in-older-adults-for-catch-up-cohorts-in-england-march-2025#data-sources-and-methodology (accessed on 11 April 2025).
28. UK Health Security Agency. Seasonal Influenza Vaccine Uptake in GP Patients: Monthly Data, 2024 to 2025. Available online: https://www.gov.uk/government/statistics/seasonal-influenza-vaccine-uptake-in-gp-patients-monthly-data-2024-to-2025 (accessed on 11 April 2025).
29. NHS England. Flu vaccinations. Weekly data. Available online: https://www.england.nhs.uk/statistics/statistical-work-areas/flu-vaccinations/ (accessed on 11 April 2025).

30. Wilkinson, T.; Beaver, S.; Macartney, M.; McArthur, E.; Yadav, V.; Lied-Lied, A. Burden of respiratory syncytial virus in adults in the United Kingdom: a systematic literature review and gap analysis. *Influenza Other Respir Viruses* **2023**, *17*, e13188, doi:10.1111/irv.13188.
31. Herbert, K.; Cabling, M.; Morris, S.; Olumogba, F.; Stockwell, S.; Todsen, A.L.; Sussex, J. *The Burden of Respiratory Syncytial Virus in Adults in the UK*; RAND Corporation: Santa Monica, CA, 2024.
32. Johannesen, C.K.; van Wijhe, M.; Tong, S.; Fernández, L.V.; Heikkinen, T.; van Boven, M.; Wang, X.; Bøås, H.; Li, Y.; Campbell, H.; et al. Age-specific Estimates of respiratory syncytial virus-associated hospitalizations in 6 European countries: a time series analysis. *J Infect Dis* **2022**, *226*, S29-s37, doi:10.1093/infdis/jiac150.
33. Howa, A.C.; Zhu, Y.; Wyatt, D.; Markus, T.; Chappell, J.D.; Halasa, N.; Trabue, C.H.; Schaffner, W.; Grijalva, C.G.; Talbot, H.K. Estimating the undetected burden of respiratory syncytial virus hospitalizations in adults through capture-recapture methods. *Influenza Other Respir Viruses* **2024**, *18*, e13299, doi:10.1111/irv.13299.
34. Sharp, A.; Minaji, M.; Panagiotopoulos, N.; Reeves, R.; Charlett, A.; Pebody, R. Estimating the burden of adult hospital admissions due to RSV and other respiratory pathogens in England. *Influenza Other Respir Viruses* **2022**, *16*, 125-131, doi:10.1111/irv.12910.
35. Hodgson, D.; Wilkins, N.; van Leeuwen, E.; Watson, C.H.; Crofts, J.; Flasche, S.; Jit, M.; Atkins, K.E. Protecting infants against RSV disease: an impact and cost-effectiveness comparison of long-acting monoclonal antibodies and maternal vaccination. *Lancet Reg Health Eur* **2024**, *38*, 100829, doi:10.1016/j.lanepe.2023.100829.
36. Zhang, T.; Reeves, R.M.; Ma, S.; Miao, Y.; Sun, S.; Orrico-Sánchez, A.; Panning, M.; Urchueguía-Fornes, A.; Vuichard-Gysin, D.; Nair, H.; et al. Estimating the respiratory syncytial virus-associated hospitalisation burden in older adults in European countries: a systematic analysis. *BMC Med* **2025**, *23*, 453, doi:10.1186/s12916-025-04249-x.
